# Toward Self‐Supported Bifunctional Air Electrodes for Flexible Solid‐State Zn–Air Batteries

**DOI:** 10.1002/smsc.202300066

**Published:** 2023-09-17

**Authors:** Xixi Wang, Lei Xu, Chuan Zhou, Ngie Hing Wong, Jaka Sunarso, Ran Ran, Wei Zhou, Zongping Shao

**Affiliations:** ^1^ State Key Laboratory of Materials-Oriented Chemical Engineering College of Chemical Engineering Nanjing Tech University Nanjing 210009 China; ^2^ Research Centre for Sustainable Technologies Faculty of Engineering, Computing, and Science Swinburne University of Technology Jalan Simpang Tiga Kuching Sarawak 93350 Malaysia; ^3^ Suzhou Laboratory Suzhou 215000 China; ^4^ Department of Chemical Engineering Curtin University Perth 6845 Australia

**Keywords:** flexible solid-state Zn–air batteries, oxygen reduction and evolution reaction, self-supported bifunctional air electrodes, synthesis strategies, wearable electronic devices

## Abstract

The demand for flexibility and rechargeability in tandem with high energy density, reliability, and safety in energy‐storage devices to power wearable electronics has translated to significant advances in flexible solid‐state Zn–air batteries (FSZABs) technology. FSZABs using self‐supported bifunctional air electrodes are currently one of the most attractive alternatives to Li‐ion battery technology for next‐generation wearable electronics. Unlike the conventional powder‐based air electrodes, self‐supported bifunctional air electrodes offer higher electron‐transfer rate, larger specific surface area (and catalyst–reactant–product interfacial contact area), mechanical flexibility, and better operational robustness. To realize their potential nonetheless, self‐supported bifunctional air electrodes should have high and stable bifunctional catalytic activity, low cost, and environmental compatibility. This review first summarizes the three typical configurations and working principles of FSZABs. Then, significant development of self‐supported bifunctional air electrodes for FSZABs and efficient synthesis strategies are emphasized. The review concludes by providing perspectives on how to further improve the electrochemical performance of FSZABs and their suitability for next‐generation wearable electronic devices.

## Graphical abstract

The recent progress of self‐supported bifunctional air electrodes for flexible solid‐state Zn–air batteries is summarized, including metal‐free carbon materials, transition metals/conductive substrates, transition metal compounds/conductive substrates, and others. The synthesis strategies for self‐supported air electrodes are then discussed. Finally, a perspective on the development of flexible solid‐state Zn–air batteries in wearable electronic devices is provided.

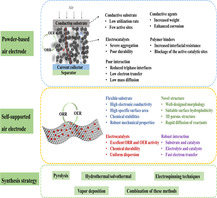

## 1 Introduction

Wearable electronic devices require both flexibility and rechargeability in parallel with high energy density, reliability, and safety. Lithium (Li)‐ion batteries, which are widely available in aqueous‐based batteries form, have become one of the most mature battery technologies, given their energy effectiveness, durability, and wide voltage window. However, they are expensive and have relatively low energy density and safety reliability, thus hindering their practical applications for advanced electronic devices, which generally require extended life cycle.^[^ ^1^ ^]^ A more attractive alternative to Li‐ion batteries, i.e., flexible solid‐state Zn–air batteries (FSZABs), has emerged, which is considered more suitable to power next‐generation wearable electronics given their excellent mechanical flexibility, easy accessibility, low cost, and environmental compatibility.^[^ ^2^ ^]^

ZABs typically comprise an air cathode, Zn anode, and conductive electrolyte. Apart from the Zn anode and electrolytes,^[^ ^3^ ^]^ recent research studies have confirmed that air electrodes are one of the vital components in enhancing FSZABs’ performance, especially in terms of their activities, superior durability, and flexibility. The sluggish reaction kinetics during charging–discharging in air electrodes is currently still the primary obstacle to obtain high FSZABs’ performance, which are intimately related with their oxygen reduction reaction (ORR) and oxygen evolution reaction (OER).^[^ ^4^ ^]^ Despite the presence of benchmark ORR material such as Pt and OER material such as RuO_2_/IrO_2_, their application in FSZABs is not practical given their high cost, poor durability, and low bifunctional activities.^[^ ^5^ ^]^ Several attempts have been made for improving air electrodes by coating powder electrocatalysts on the conductive substrates. However, adding conductive agents and polymer binders may decrease the battery performance during charging–discharging.^[^ ^6^ ^]^ Hence, using self‐supported bifunctional air electrodes for FSZABs is more attractive to obtain high electrochemical activity and stability as well as flexibility,^[^ ^7^ ^]^ which come from maintaining their sizeable interfacial contact area, high specific surface area, fast electron‐transport paths, and superior bifunctionality and flexibility.^[^ ^8^ ^]^ Therefore, developing self‐supported bifunctional air electrodes by growing the active catalysts directly on the conductive substrate's surface is of interest to achieve high‐performance FSZABs.

**Figure 1:**
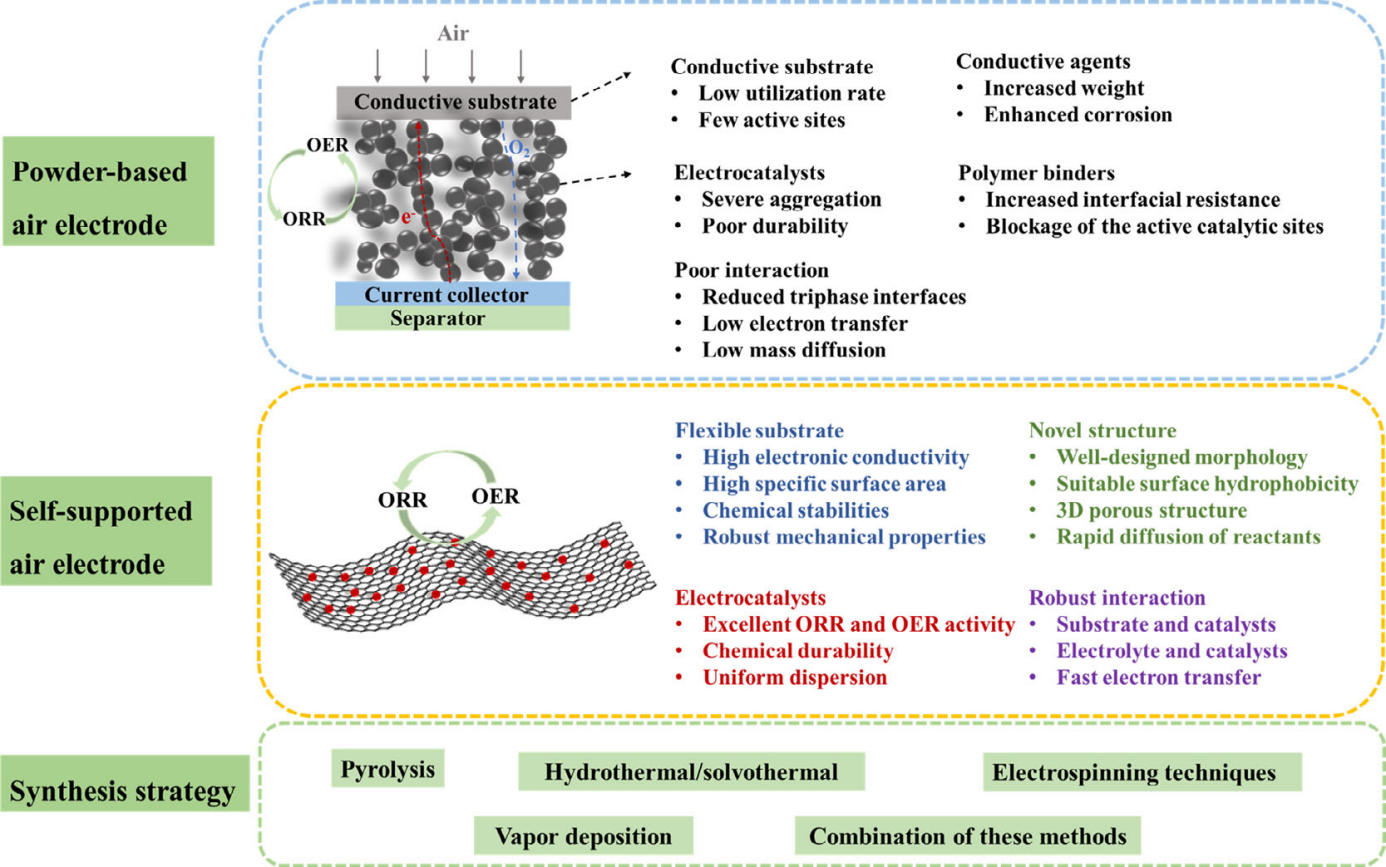
Summary of powder‐based air electrodes, self‐supported air electrodes, and synthesis strategies of self‐supported air electrodes for ZABs.

In this review, we focus on the self‐supported bifunctional air electrodes for FSZABs (**Figure** **1**). We first discuss the battery configuration and working principles. Then, we discuss the ORR and OER mechanisms and their performance evaluation criteria. Next, we summarize the advances toward powder catalysts and the drawbacks of powder catalysts in rechargeable ZABs. We mainly highlight a detailed overview summary of self‐supported bifunctional air electrodes in FSZABs, categorizing them into metal‐free carbon materials, transition metals/conductive substrates, transition metal compounds/conductive substrates, and other air electrodes. Before concluding this review with our perspectives on the FSZABs’ development for wearable electronic devices, we also discuss different synthesis strategies for self‐supported air electrodes in detail.

## 2 ZAB Background

### 2.1 Battery Configuration

**Figure 2:**
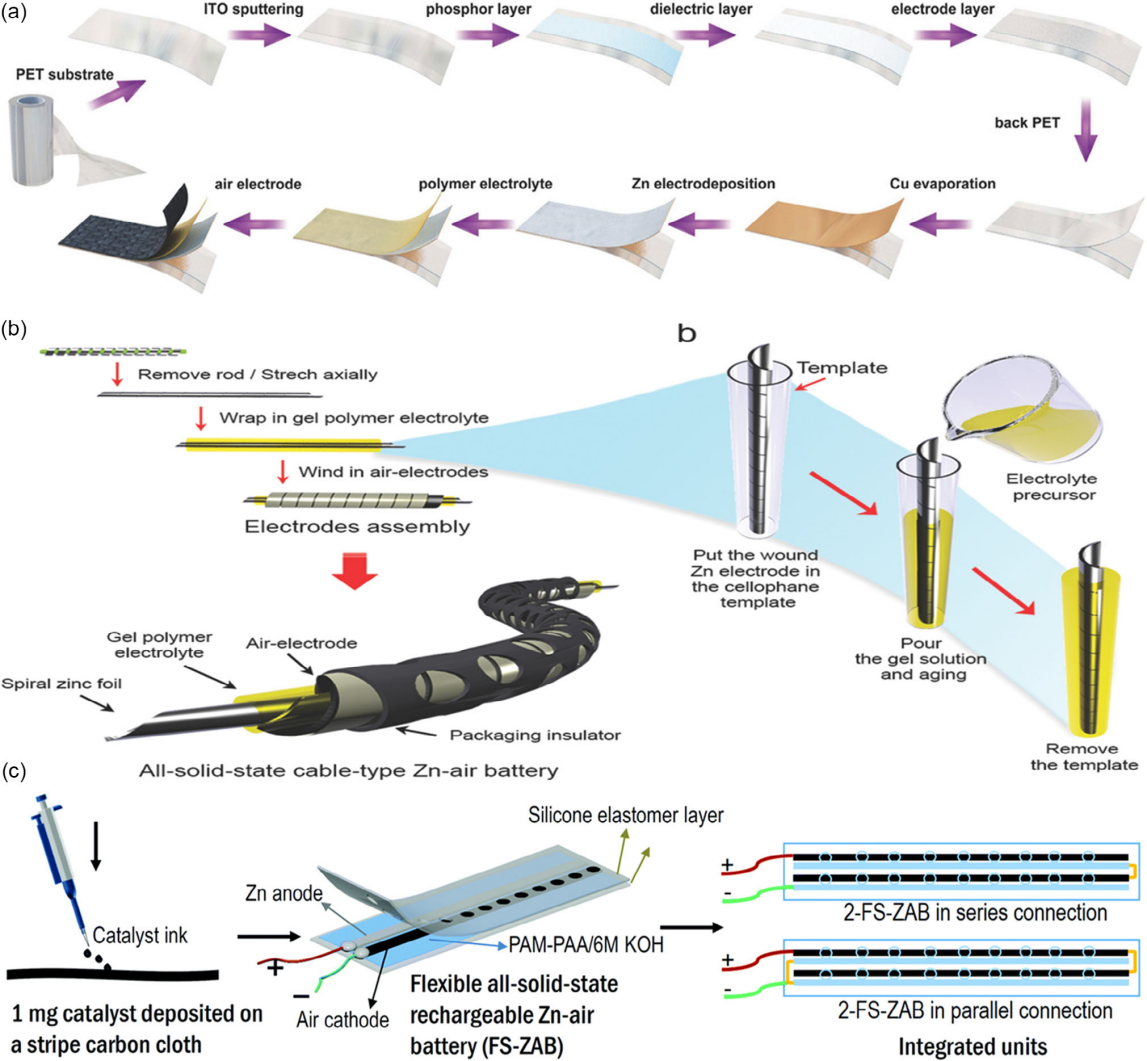
a–c) Schematic illustrations of the fabrication procedures for sandwich (a), cable (b), and coplanar (c) types of ZABs. a) Reproduced with permission.^[^ ^64^ ^]^ Copyright 2017, Wiley‐VCH. b) Reproduced with permission.^[^ ^11^ ^]^ Copyright 2014, Wiley‐VCH. c) Reproduced with permission.^[13b]^ Copyright 2020, Royal Society of Chemistry.

Besides high energy efficiency and outstanding mechanical properties under various formation states, battery configuration also occupies a vital position in determining the electrocatalytic performance of these FSZABs and wearable electronic devices.^[^ ^9^ ^]^ Here, we focus on three types of FSZABs configurations, i.e., sandwich, cable, and coplanar. Sandwich‐type ZABs are the most popular configuration for FSZABs. They comprise a Zn anode, solid‐state electrolyte membrane, and air cathode stacked side by side (**Figure** **2**a). The sandwich‐like structure endows excellent bending, twisting, and stretching proprieties, which well suits small wearable applications.^[^ ^10^ ^]^ Compared with sandwich‐type ZABs, the cable‐type ZABs exhibit more outstanding mechanical properties and higher volumetric energy density since their appearance in 2014.^[^ ^11^ ^]^ For cable‐type ZABs, the Zn anode comprises a membrane‐wrapped electrolyte encapsulated with a breathable heat shrink tube (Figure 2b). Cable‐type ZABs can be promising upcoming wearable and compact energy‐storage devices because they can be part of integrated fashion design for powering wearable electronics.^[^ ^11^, ^12^ ^]^ Finally, coplanar‐type ZABs, which have been recently explored, is also compatible for wearable electronic devices application. The air cathode and ZN anode are purposely arranged in a plane and on the same side as the electrode for coplanar‐type ZABs (Figure 2c). Such a configuration can provide robust flexibility and coplanar integrability, preventing the solid electrolyte film to be detached from the electrode when it deforms under repeating bending.^[^ ^13^ ^]^

### 2.2 Working Principles

A typical ZAB comprises an air cathode, Zn anode, and conductive electrolyte. Its working principles can be divided into discharging process and charging process (Equation (1)). During the discharging process, the anode Zn is oxidized (Equation (2)) and finally decomposes into ZnO (Equation (3)), while the oxygen in cathode is reduced to OH^-^ ions (Equation (4)). Moreover, ZnO can be reduced to Zn metal via the reversible reaction in the Zn anode. And OH^−^ ions can be oxidized to O_2_ in the air cathode during the charging process.^[^ ^14^ ^]^

(1) $${\text{Overall reaction: 2Zn + O}}_{\text{2 }}↔{\text{ 2ZnO (}E}_{\text{eq}}\text{ = 1.65}\text{ V vs SHE)}$$

(2) $${\text{Zn anode: Zn + 4OH}}^{‐}\;↔\text{ Zn}{\left(\text{OH}\right)}_{4}^{\text{2‐}}{\text{ + 2e}}^{‐}$$

(3) $${\text{Zn(OH)}}_{4}^{\text{2‐}}\;↔{\text{ ZnO + H}}_{2}{\text{O + 2OH}}^{‐}$$

(4) $$\text{Air cathode}:{\text{ O}}_{2}{\text{ + 2H}}_{2}{\text{O + 4e}}^{‐}\;↔{\text{ 4OH}}^{‐}$$

Besides the aforementioned electrochemical reactions, the theoretical and working voltages are the other critical factors affecting the performance of ZABs. Generally, ZABs possess 1.65 and 1.2 V of theoretical and working voltages, respectively.[^1^, ^15^] However, a high potential gap, low round‐trip energy efficiency, and poor long‐term durability may significantly constrain the overall performance of the rechargeable ZABs. Besides that, the mechanical properties of ZABs are also essential for the outstanding electrochemical performances of wearable and power devices.^[^ ^16^ ^]^ Therefore, many researchers have devoted their studies to air cathodes, Zn anodes, and electrolytes, improving the ZAB's rechargeable performance and mechanical flexibility properties.

Most reported ZABs used pure Zn plates, Zn foils, and Zn wires directly as the Zn anodes, which may lead to low interfacial contact area and utilization.[^5^, ^17^] Several attempts have been performed recently to overcome these setbacks. For example, Liu and co‐workers introduced anode additives (metal elements and relative oxides), modified Zn morphology, and electrodeposited Zn particles on flexible carbon cloth.^[^ ^18^ ^]^ Another challenge, such as the evaporation and leakage of the liquid electrolytes when using KOH alkaline solution (6 m), can degrade the lifetime of rechargeable ZABs, and packaging limitation of liquid electrolytes also causes the excessive weight of batteries, which could not meet the requirements of flexible devices.^[^ ^19^ ^]^ Hence, other types of alkaline gel electrolytes have been considered, which could conduct ions, separate the electrodes, and support the flexible structures. For example, poly(acrylic acid) (PAA), poly(vinyl alcohol) (PVA), poly(ethylene oxide) (PEO), and polyacrylamide (PAM) are currently some of the promising solid electrolytes for FSZABs, given their high conductivity, suitable water retention, and desired mechanical properties.^[^ ^20^ ^]^

On the other hand, air cathode preparation methods can be time‐consuming, complicated, and ineffective.^[^ ^21^ ^]^ This is because the traditional air cathode comprises powder catalysts, conductive agents, polymer binders, and conductive substrates for forming a conductive network. Conductive agents (acetylene or super P carbon black) addition to the battery generally leads to increased weight and enhanced corrosion during the charging, which can weaken its performance.^[^ ^22^ ^]^ Similarly, when the polymer binders (Nafion and PTFE) are added to the battery, the interfacial resistance typically increases due to the blockage of the active catalytic sites, which reduces the battery's lifetime.^[^ ^23^ ^]^

Given the aforementioned explanations, the subsequent discussions will focus onto the self‐supported air electrodes for the FSZABs. The self‐supported air electrodes should possess several advantages as follows: 1) their use avoids the need to add more conductive agents and polymer binders, giving better scalability; 2) these electrodes possess larger interfacial contact area between the catalysts and substrates with high charge transfer rate, excellent flexibility, and stability; and finally 3) these electrodes allow better catalysts dispersion and thus, more catalytically active sites. Usually, we construct a self‐supported air electrode by directly growing catalysts on conductive substrates with controlled nanostructure and morphology to meet the requirements of the latest intelligent wearable technology and wireless communication applications.^[^ ^24^ ^]^

The substrates for FSZABs should possess excellent flexibility, air permeability, high electronic conductivity, and robust mechanical performance when undergoing repetitive shape deformation. In general, metal and carbon‐based substrates are usually chosen as the conductive substrates for FSZABs. Metal‐based substrates have the advantages of high conductivity, mechanical strength, and stability, which possess satisfactory mechanical properties when FSZABs are subjected to complex and repeated deformation. Metal‐based substrates mainly include metal foils, metal meshes, and metal foams.^[^ ^25^ ^]^ First, the solid structure of metal foils will hinder the continuous supply of oxygen and reduce the battery performance, so it is rarely used in FSZABs. Second, metal meshes and metal foams have high conductivity, large porosity, and high mechanical strength, which benefits the diffusion of reactants and oxygen to active sites, thus improving battery performance. However, metal‐based substrates are generally less stable due to fatigue during the bending, twisting, and stretching process. In addition, the high weight of the metal‐based substrates increases the mass weight of the battery, which reduces the actual energy density of the battery.

In fact, researchers tend to use carbon‐based substrates with light weight, excellent mechanical flexibility, high conductivity, excellent deformability, easy fabrication, and high porosity, including carbon paper (CP), carbon cloth (CC), carbon nanofiber film (CNF), carbon nanotube film (CNT), carbon fiber paper (CFP), and graphene oxide paper (GO).^[^ ^26^ ^]^ First, the available commercial CC, CP, and CFP substrates have been applied widely in FSZABs, but the performance is not very ideal. Second, the CNF‐based films prepared by electrospinning technology shows the advantages of adjustable components and excellent mechanical flexibility, which shows improved battery performance compared to commercial carbon materials. At the same time, graphene can also promote the battery performance due to its high specific surface area, excellent conductivity, and tolerance under severe working conditions. More importantly, CNTs have been considered as the most ideal substrates for self‐supported air electrodes due to their large surface area, outstanding mechanical strength, and abundant accessible pathways for mass transportation, thus possessing superior battery performance generally.

### 2.3 ORR and OER Mechanisms

ORR and OER with slow oxygen electrochemical reaction kinetics in the air electrodes can hinder the performance of FSZABs. Equation (5)–(10) represent the ORR that involves complicated reactions with multiple‐electron transfer at the air cathode, where * represents the active site on the catalyst surface. These complex reactions are classified into two‐electron (Equation (5) and (6)) and four‐electron (Equation (7)–(10)) transfer steps, respectively.^[^ ^27^ ^]^ Compared to the four‐electron transfer steps, the generation of peroxides in the former transfer steps usually leads to inadequate energy and stability. Typically, the adsorption–desorption behavior of HOO*, O*, and HO* in the four‐electron transfer steps determines the electrochemical activity of the catalysts for ORR.^[1a]^ Researchers usually prefer the four‐electron transfer route of ORR for FSZABs.

(5) $$O_{2}+H_{2}{O+\text{2e}}^{-}={\text{HO}}_{2}^{-}+{\text{OH}}^{-}$$

(6) $${\text{HO}}_{2}^{-}+H_{2}{O+\text{2e}}^{-}={\text{3OH}}^{-}$$

(7)

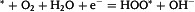

(8) $${\text{HOO}}^{*}+e^{-}=O^{*}+{\text{OH}}^{-}$$

(9) $$O^{*}+H_{2}{O+e}^{-}={\text{HO}}^{*}+{\text{OH}}^{-}$$

(10)

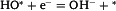




**Figure 3:**
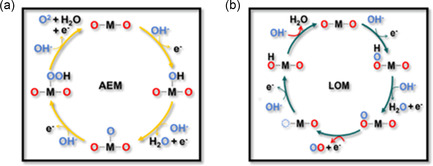
a,b) Schematic descriptions of AEM (a) and LOM (b) for the OER. Reproduced with permission.^[^ ^90^ ^]^ Copyright 2023, Wiley‐VCH.

The conventional adsorbate evolution mechanism (AEM) and the lattice‐oxygen‐mediated mechanism (LOM) for OER are depicted in **Figure** **3**. First, the reaction process for the AEM is similar to ORR but in the opposite order (Figure 3a). A proportional relationship exists between the adsorption energies of the aforementioned oxygen‐containing intermediates, resulting in a minimum 0.37 V overpotential for this mechanism.^[^ ^28^ ^]^ According to Sabatier's theory, the oxidation of HO* becomes the rate‐determining step at low binding energy. In contrast, the formation of HOO* becomes the rate‐determining step when the binding energy is high.^[^ ^29^ ^]^ Unlike the AEM, the LOM can avoid the formation of HOO* (Figure 3b). The lattice oxygen atom reacts with the adsorbed oxygen or combines with another lattice oxygen atom to form O—O bonds, thus bypassing the aforementioned overpotential limitation (0.37 V) caused by the proportional relationship between HO* and HOO*.^[^ ^28^, ^30^ ^]^

Based on these mechanisms, the redox ability of metal and oxygen ions during the OER processes determines which reaction mechanism occurred on the catalyst's surface. When the electron‐transfer ability of metal ions becomes more substantial, the OER proceeds with the AEM mechanism, and the oxygen‐containing reaction intermediate determines the overpotential. When the lattice oxygen atom is activated for the OER, the deprotonation ability of the oxygen‐containing intermediate determines the OER rate.^[^ ^29^, ^31^ ^]^

### 2.4 Evaluation Criteria

Here, we listed the critical criteria for assessing the electrochemical performance of ORR: 1) the onset potential (*E*_onset_), 2) half‐wave potential (*E*_1/2_), 3) limiting current density (*J*_L_), 4) Tafel slope (smaller Tafel slope value indicates more favorable reaction kinetics), 5) electron transfer number (*n*), 6) hydrogen peroxide yield, 7) mass activity (MA), 8) intrinsic activity (SA), and 9) stability. As for the OER, the critical parameters to assess the OER electrochemical activity are 1) the overpotential at a current density of 10 mA cm^−2^ (*η*), 2) charge transfer resistance (*R*_ct_), 3) current density (*J*), 4) Tafel slope, 5) mass activity (MA), 6) intrinsic activity (SA), and 7) stability.

Then, to analyze the bifunctional electrochemical performance, the potential difference Δ*E* between the overpotential of OER and the half‐wave potential of ORR generally can be calculated, where a smaller Δ*E* represents a better bifunctional electrochemical activity.^[^ ^32^ ^]^ Finally, to compare the performance of different catalysts in air electrodes for ZABs, the critical parameters are: 1) charge–discharge potential gap (Δ*E*), 2) round‐trip efficiency, 3) specific capacity, 4) energy density, 5) open‐circuit voltage (OCV), 6) peak power density, and 7) charge–discharge cycling stability.^[8a]^

## 3 Self‐Supported Air Electrodes for FSZABs

Traditional air electrodes using powder electrocatalysts can improve the electrochemical performance and durability of ZABs. **Table** **1** summarizes the performances of recently developed powder catalysts for aqueous ZABs and FSZABs. These powder catalysts can be classified into 1) precious metal‐based catalysts, 2) transition metal‐based catalysts (metal alloys, oxides, hydroxides, nitrides, and phosphides), and 3) metal‐free carbon catalysts. The performance of powder electrocatalysts can be modified through component modulation, structural modulation, size adjustment, morphology design, and composite construction, which show improved catalytic performance of aqueous ZABs. However, the evaporation and leakage of liquid electrolytes will inevitably decrease the lifetime of ZABs, and packaging limitation of liquid electrolytes also causes the excessive weight of batteries, which could not meet the requirements of wearable electronic devices. In contrast, the solid electrolyte in FSZABs can conduct ions, separate the electrodes, and support the flexible structures, which simplify the production process of batteries. In addition, the FSZABs possess higher safety, environmental friendliness, and flexibility, which show great potential for commercialization in flexible devices compared with aqueous ZABs. Obviously, the ionic conductivity of solid electrolyte is much lower than that of aqueous electrolyte, thus leading to lower battery performance of FSZABs than that of aqueous Zn–air batteries.

**Table 1:** The OER, ORR (in a 0.1 m KOH), and catalytic performances of recently developed powder catalysts for aqueous ZABs and FSZABs

Powder catalyst/substrate	Battery configuration	OER@*η* [V]/ORR@*E*_1/2_ [V]	Δ*E* [V]/initial round‐trip efficiency	Battery performance (peak power density; specific capacity; energy density; OCV; charge–discharge)	References
CoNi@NCNT/Ni foam	Aqueous	0.33/0.70	0.78 V/58.9%	127 mW cm^−2^@200 mA cm^−2^; 655 mAh g^−1^@5 mA cm^−2^; 845 Wh kg^−1^@5 mA cm^−2^; 1.40 V; @5 mA cm^−2^ for 90 h	[25c]
Co_3_O_4_–C–NA/Ni foam	Aqueous	0.31/0.83	0.93/53.4%	118 mW cm^−2^@248 mA cm^−2^; 1.42 V; @30 mA cm^−2^ for 180 cycles	[25d]
HNG‐900/CC	Aqueous	0.46/0.78	0.78/58.5%	68 mW cm^−2^; 790 mAh g^−1^@5 mA cm^−2^; @2 mA cm^−2^ for 280 h	[91]
NC–NH_3_ + COMT@N/Ni foam	Aqueous	0.55/0.82	0.70/64.5%	@10 mA cm^−2^ for 800 h	[92]
EN‐PCNS/CC	Sandwich	0.58/0.82	0.82/59.4%	139 mW cm^−2^@158.2 mA cm^−2^; 766 mAh g^−1^@5 mA cm^−2^; 1.47 V; @0.5 mA cm^−2^ for 600 min	[93]
N/E‐HPC‐900/CFP	Aqueous	0.38/0.85	1.20/48.9%	192.7 mW cm^−2^@390.4 mA cm^−2^; 801 mAh g^−1^@10 mA cm^−2^; 955 Wh kg^−1^@10 mA cm^−2^; 1.49 V; @10 mA cm^−2^ for 110 h	[94]
	Aqueous	0.38/0.85	0.77/58%	689 Wh kg^−1^@10 mA cm^−2^; 749 mAh g^−1^@10 mA cm^−2^; 36.2 mW cm^−2^@47.8 mA cm^−2^; 1.34 V; @10 mA cm^−2^ for 80 h	
NPMC‐1000/CP	Aqueous	0.65/0.85	0.94/57.1%	55 mW cm^−2^@70 mA cm^−2^; 735 mAh g^−1^@5 mA cm^−2^; 835 Wh kg^−1^@5 mA cm^−2^; 1.48 V; @2 mA cm^−2^ for 100 h	[95]
NPCS‐900/CC	Aqueous	0.42/0.79	0.90/56.7%	79 mW cm^−2^@122 mA cm^−2^; 684 mAh g^−1^@2 mA cm^−2^; 848 Wh kg^−1^@2 mA cm^−2^; 1.39 V; @20 mA cm^−2^ for 40 h	[96]
	Sandwich		0.80/60%	55 mW cm^−2^@50 mA cm^−2^; 642 mAh g^−1^@1 mA cm^−2^; 1.40 V; @1 mA cm^−2^ for 30 h	
NOGB‐800/CC	Aqueous	0.40/0.84	1.10/42.6%	111.9 mW cm^−2^@178 mA cm^−2^; @10 mA cm^−2^ for 30 h	[97]
NPSCSs/CFP	Aqueous	0.43/0.83	0.54/72.8%	120.5 mW cm^−2^@220 mA cm^−2^; @10 mA cm^−2^ for 45 h	[98]
C‐MOF‐C2‐900/Ni foam	Aqueous	0.35/0.82	0.53/70.7%	105 mW cm^−2^@120 mA cm^−2^; 768 mAh g^−1^@5 mA cm^−2^; 1.46 V; @10 mA cm^−2^ for 40 h	[99]
(Zn, Co)/NC/CC	Aqueous	0.40/0.87	0.57/62.8%	186 mW cm^−2^@301 mA cm^−2^; 807 mAh g^−1^@50 mA cm^−2^; @50 mA cm^−2^ for 60 h	[100]
2D Fe‐N‐C/Ni foam	Aqueous	0.46/0.91	0.45/72.7%	156 mW cm^−2^; 798 mAh g^−1^@30 mA cm^−2^; 1.47 V; @5 mA cm^−2^ for 120 h	[101]
Fe‐NSDC/Ni foam	Aqueous	0.41/0.84	0.43/75.6%	225.1 mW cm^−2^; 740.8 mAh g^−1^@4 mA cm^−2^; 1.53 V; @4 mA cm^−2^ for 67 h	[102]
	Sandwich		–	@2 mA cm^−2^ for 13 800 s (FSZABs)	
FeCo–N_*x*_–CN‐30/CC	Aqueous	0.44/0.89	0.75/62.7%	150 mW cm^−2^@261 mA cm^−2^; 1.4 V; @10 mA cm^−2^ for 44 h	[103]
α‐MnO_2_‐120/CC	Aqueous	0.76/0.85	–	240 mW cm^−2^; 731 mAh g^−1^@10 mA cm^−2^; 1.27 V; @10 mA cm^−2^ for 15 h	[104]
Ag‐MnO_2_/CP	Aqueous	0.64/0.67	1.09/52.4%	273.2 mW cm^−2^@481 mA cm^−2^; 683.1 mAh g^−1^@10 mA cm^−2^; 915.4 Wh kg^−1^@10 mA cm^−2^; @10 mA cm^−2^ for 107 h	[105]
NiFe‐LDHs@MnO_2_/Ni foam	Aqueous	–	0.97/53.2%	80 mW cm^−2^@115.6 mA cm^−2^; 1.41 V; @25 mA cm^−2^ for 42 h	[106]
TiC/α‐MnO_2_/NW/SS	Aqueous	0.41/0.78	0.86/55.7%	161.3 mW cm^−2^@246.7 mA cm^−2^; @20 mA cm^−2^ for 5,000 min	[107]
	Sandwich		0.72/61.3%	61.2 mW cm^−2^@105.8 mA cm^−2^; 1.312 V; @5 mA cm^−2^ for 600 h (FSZABs)	
Co_3_O_4_/Co–N@NMC/CFP	Aqueous	0.35/0.79	0.744/65.5%	122 mW cm^−2^; 505 mAh g^−1^@25 mA cm^−2^; 1.46 V; @25 mA cm^−2^ for 36 h	[108]
NiCo–air/CC	Aqueous	0.44/0.74	0.95/53.9%	102.08 mW cm^−2^@178 mA cm^−2^; 1.38 V; @10 mA cm^−2^ for 29 h	[109]
NiCo_2.14_8O_4_ PNSs/CFP	Aqueous	0.19/0.65	0.84/50.6%	83 mW cm^−2^@150 mA cm^−2^; 700 Wh kg^−1^@1 mA cm^−2^; 1.46 V; @5 mA cm^−2^ for 120 h	[110]
	Cable		–	1.33 V; @1 mA cm^−2^ for 10 h (FSZABs)	
CoMn_2_O_4_ 3DOM/CC	Aqueous	0.56/0.74	0.72/58.7%	101.6 mW cm^−2^@190 mA cm^−2^; 1440 mAh g^−1^; 1.42 V	[111]
NiCo_2_O_4_@FeNi LDH/Ni foam	Aqueous	0.44/0.78	0.65/65.7%	130 mW cm^−2^@ 200 mA cm^−2^; 810 mAh g^−1^@10 mA cm^−2^; 1.40 V; @10 mA cm^−2^ for 90 h	[112]
Mn_0.5_Ni_0.5_Co_2_O_4_/CC	Aqueous	0.40/0.76	0.77/60.9%	117 mW cm^−2^@175 mA cm^−2^; 1587 mAh g^−1^@3 mA cm^−2^; 1.38 V; @10 mA cm^−2^ for 21 h	[113]
LaCoO_3_Ce‐5.6%/CC	Aqueous	0.45/0.72	0.81/59.7%	60 mW cm^−2^@85 mA cm^−2^; 783 mAh g^−1^@5 mA cm^−2^; 1.426 V; @2 mA cm^−2^ for 160 h	[114]
	Coin		0.63/65%	31 mW cm^−2^@64 mA cm^−2^; 1.33 V; @2 mA cm^−2^ for 7.4 h	
S_5.84%_‐LCO/CP	Aqueous	0.36/0.70	0.73/62.6%	92 mW cm^−2^@144 mA cm^−2^; 747 mAh g^−1^@5 mA cm^−2^; 1.47 V; @5 mA cm^−2^ for 120 h	[115]
	Sandwich		0.85/57.5%	1.38 V; @4 mA cm^−2^ for 16 h (FSZABs)	
CMO/S‐300/CP	Aqueous	0.47/0.76	0.67/64.8%	152 mW cm^−2^@128 mA cm^−2^; @5 mA cm^−2^ for 120 cycles	[116]
	Sandwich		0.96/53.6%	1.32 V; @1 mA cm^−2^ for 10 h (FSZABs)	
20%GDC‐PBC/CFP	Aqueous	0.41/0.72	0.82/57.6%	207 mW cm^−2^@320 mA cm^−2^; @1 mA cm^−2^ for 10 h	[117]
CoP‐PBSCF/CC	Aqueous	0.38/0.75	0.89/58.4%	138 mW cm^−2^; @10 mA cm^−2^ for 32 h	[118]
Co_3_O_4_@LaCoO_3_/CFP	Aqueous	0.32/0.67	0.69/65.5%	140 mW cm^−2^@285 mA cm^−2^; 785 mAh g^−1^@5 mA cm^−2^; 1.46 V; @2 mA cm^−2^ for 185 h	[119]
Co‐MOF/LC‐0.5/CC	Aqueous	0.33/‐	0.67/66.5%	126 mW cm^−2^@107 mA cm^−2^; 1.44 V; @2 mA cm^−2^ for 140 h	[120]
Co_2_FeO_4_/NCNTs/CC	Aqueous	0.43/0.80	0.89/56.9%	90.68 mW cm^−2^@170 mA cm^−2^; 1.43 V; @50 mA cm^−2^ for 100 h	[121]
LSM30/CC	Aqueous	0.49/0.71	0.793/59.8%	181.4 mW cm^−2^@260 mA cm^−2^; @10 mA cm^−2^ for 100 cycles	[122]
LTFO‐C/CFP	Aqueous	0.54/0.85	0.57/69%	440 mAh g^−1^; 60 cycles	[123]
MnCo_2_O_4_/NCNTs/CFP	Aqueous	0.37/0.76	1.11/53.0%	74.63 mW cm^−2^@140 mA cm^−2^; 827 mAh g^−1^@100 mA cm^−2^; 1.48 V; @5 mA cm^−2^ for 300 cycles	[124]
	Sandwich		0.84/59.2%	@5 mA cm^−2^ for 20 cycles (FSZABs)	
P‐3G/Ni foam	Aqueous	0.32/0.82	0.88/56.1%	128.5 mW cm^−2^@250 mA cm^−2^; @10 mA cm^−2^ for 110 cycles	[125]
NiCo/NLG‐270/CP	Aqueous	0.34/0.82	0.80/60.9%	103 mW cm^−2^@140 mA cm^−2^; 403 mAh g^−1^@10 mA cm^−2^; 505 Wh kg^−1^@10 mA cm^−2^; 1.49 V; @20 mA cm^−2^ for 14 h	[126]
NCNT/Co_0.51_Mn_0.49_O/Ni foam	Aqueous	0.34/0.84	0.57/65.7%	581 mAh g^−1^@7 mA cm^−2^; 695 Wh kg^−1^@7 mA cm^−2^; @7 mA cm^−2^ for 44 000 s	[127]
CoO_0.87_S_0.13_/GN/cellulose membrane	Aqueous	0.42/0.83	0.76/60.4%	709 mAh g^−1^@10 mA cm^−2^; 857.9 Wh kg^−1^@10 mA cm^−2^; 1.43 V; @20 mA cm^−2^ for 300 h	[128]
Co_9_S_8_/NSC/CP	Aqueous	0.41/0.88	0.91/55.1%	@10 mA cm^−2^ for 5000 min	[129]
Ni_3_FeN/NRGO/CFP	Aqueous	0.40/0.84	0.77/60.3%	@10 mA cm^−2^ for 30 h	[130]
CC‐A‐N@Co–NNCT/CC	Aqueous	0.40/0.80	0.6/67.4%	172 mW cm^−2^@270 mA cm^−2^; @2 mA cm^−2^ for 100 h	[131]
	Sandwich		0.67/64.4%	44 mW cm^−2^@40 mA cm^−2^; 1.42 V; @2 mA cm^−2^ for 3 h (FSZABs)	
NC–Co/CoN_*x*_/CC	Cable	0.29/0.88	0.6/67.7%	41.5 mW cm^−2^@96.6 mA cm^−2^; 1.40 V	[132]
Fe–Co_4_N@N–C/CP	Aqueous	0.32/0.83	0.77/60.9%	105 mW cm^−2^; 806 mAh g^−1^@5 mA cm^−2^; 934 Wh kg^−1^@5 mA cm^−2^; 1.46 V; @5 mA cm^−2^ for 36 h	[133]
	Sandwich		0.74/62.1%	72 mW cm^−2^; 1.34 V; @4 mA cm^−2^ for 45 cycles (FSZABs)	
Co SA@NCF/CNF/CC	Cable	0.4/0.88	0.6/67.6%	530.2 mAh g^−1^; 1.41 V; @6.25 mA cm^−2^ for 15 h	[134]
Ce@Co_3_O_4_/CNFs/CP	Aqueous	0.38/0.81	1.11/46.6%	97.7 mW cm^−2^@221 mA cm^−2^; 1.40 V; @5 mA cm^−2^ for 50 h	[135]
SS@L‐CoO/Ni foam	Aqueous	0.32/0.8	0.65/62.3%	@3 mA cm^−2^ for 550 h and @20 mA cm^−2^ for 550 h	[136]
Co_3_O_4_/Ni foam	Aqueous	0.30/0.66	1.13/47.2%	35.7 mW cm^−2^@68 mA cm^−2^; 711 mAh g^−1^@5 mA cm^−2^; @10 mA cm^−2^ for 333 h	[137]
N‐GCNT/FeCo‐3/CFP	Sandwich	0.50/0.92	0.75 V/50.3%	97.8 mW cm^−2^; 495 mAh g^−1^@10 mA cm^−2^; 1.15 V; @100 mA cm^−2^ for 12 h	[138]
NC–Co/CoN_*x*_/CC	Cable	0.33/0.87	0.59/69.7%	104 mW cm^−2^	[139]
CoN_4_/NG/CC	Cable	0.38/0.87	0.66/65.5%	28 mW cm^−2^; @1.0 mA cm^−2^ for 5 h	[140]
CoCNTs/PNAs/CP	Aqueous	0.31/0.925	0.81/58.7%	371.6 mW cm^−2^@587 mA cm^−2^; 761.9 mAh g^−1^; 1.51 V; @5.0 mA cm^−2^ for 2000 h	[16b]
	Cable		0.59/69.7%	1.38 V; @5.0 mA cm^−2^ for 2000 min	
NdC–CoNP–NdC‐700/CC	Coplanar	0.40/0.80	0.87/57.1%	57 mW cm^−2^@103 mA cm^−2^; 771 mA h g^−1^@2 mA cm^−2^; 903 W h kg^−1^@5 mA cm^−2^; 1.42 V; @5.0 mA cm^−2^ for 50 h	[13a]
Co–Fe–S@NSRPC/CC	Coplanar	0.37/0.80	0.87/57.1%	78 mW cm^−2^; 785 mA h g^−1^@5 mA cm^−2^; 903 W h kg^−1^@5 mA cm^−2^; 1.42 V; @5.0 mA cm^−2^ for 50 h	[13b]
Fe/Fe_3_C@NdC‐NCs/CC	Coplanar	0.39/0.83	0.83/57.8%	12.76 mW cm^−2^; 736 mA h g^−1^@5 mA cm^−2^; 1.43 V; @5.0 mA cm^−2^ for 40 h	[13c]
IOSHs‐NSC‐Co_9_S_8_/CC	Coplanar	0.41/0.82	0.93/55.7%	60 mW cm^−2^; 738 mA h g^−1^@5 mA cm^−2^; 900.4 W h kg^−1^@5 mA cm^−2^; 1.41 V; @5.0 mA cm^−2^ for 35 h	[67]

More importantly, the inherent properties of powder catalysts have limited the performance improvement of FSZABs: 1) the use of polymer binder blocks the transfer of electron and mass, lowering the ORR and OER activity of catalysts, and its degradation also leads to the shedding of the catalysts; 2) the addition of conductive carbon increases the weight of electrode by 10–40 wt% and reduces the battery energy density. In addition, the corrosion of carbon during the OER process also decreases the electronic conductivity and catalytic performance; 3) the agglomeration of powder catalysts is inevitable and the contact with the conductive substrates also is insufficient; 4) the powder catalysts are easily detached from the air electrodes during the mechanical stress test; and 5) the preparation process is complex and time‐consuming. All above shortcomings eventually cause poor performance of powder‐based FSZABs, especially the durability and flexibility. As shown in Table 1, the power density of powder‐based FSZABs is generally less than 100 mW cm^−2^, and cycling stability can only lasts for about 30 h under low current density.

In this case, FSZABs using self‐supported air electrodes have attracted the attention of researchers. Compared to powder‐based FSZABs, self‐supported air electrodes do not need the additional conducting carbon and binder, thus possessing more catalytic sites and reduced interface contact resistance. In addition, the conductive substrates in self‐supported air electrode act as both current collectors and substrates for the catalyst growth, which leads to tight combination between the catalysts and substrates. Therefore, FSZABs based on self‐supported air electrodes tend to have better catalytic performance. **Table** **2** summarizes the FSZABs’ performances based on recently developed self‐supported bifunctional air electrodes, where the maximum power density and energy density can reach 232 mW cm^−2^ and 965.2 Wh kg^−1^, respectively. In addition, the battery can maintain long‐term stable operation under high current density and different bending angles, which displays superior improvement over powder‐based FSZABs. Hence, this section mainly focuses on the discussion of typical self‐supported bifunctional air electrodes, such as metal‐free carbon materials, transition metal/conductive substrates, transition metal compounds/conductive substrates, and others for FSZABs.

**Table 2:** The FSZABs performances based on recently developed self‐supported bifunctional air electrodes

Air electrodes	Battery configuration	Flexible substrate	Electrolyte	Δ*E* [V]/initial round‐trip efficiency	Battery performance (peak power density; specific capacity; energy density; OCV; charge–discharge)	References
NCNF‐1000	Sandwich	NCNFs	PVA	0.78/56.2%	378 mAh g^−1^@2 mA cm^−2^; 378 Wh kg^−1^@2 mA cm^−2^; 1.26 V; @2 mA cm^−2^ for 6 h	[37]
N, S‐CC	Sandwich	CC	PVA	1.28/42.6%	47 mW cm^−2^; 1.25 V; @5 mA cm^−2^ for 120 cycles	[131]
3D NCNT array	Sandwich	Ni foam	PVA	0.96/51.5%	356 mAh g^−1^@5 mA cm^−2^; 382 Wh kg^−1^@5 mA cm^−2^; @5 mA cm^−2^ for 9000 s	[36]
CNT@POF	Sandwich	CNTs	PVA	0.76/61.6%	22.3 mW cm^−2^; 1.39 V; @1 mA cm^−2^ for 12 cycles	[38]
CNTs–NC–CCC	sandwich	CNTs	PVA	0.42/74.1%	68 mW cm^−2^@5 mA cm^−2^; 1.35 V; 200 cycles	[39]
D‐S/N‐GLC	Sandwich	Graphene‐like carbon	A901	–	81 mW cm^−2^@168 mA cm^−2^; @5 mA cm^−2^ for 10 000 s	[41]
BNF‐LCFs	Sandwich	CNFs	PVA	0.94/53.9%	1.358 V; @1 mA cm^−2^ for 12 h	[42]
MPZ‐CC@CNT	Sandwich	CNTs	PVA	0.97/52.9%	860 mAh g^−1^@25 mA cm^−2^; 946 Wh kg^−1^@25 mA cm^−2^; 1.47 V; @50 mA cm^−2^ for 600 h	[46]
CC‐A‐N@Co–NCNT	Sandwich	NCNTs	PVA and KOH	0.67/64.4%	44 mW cm^−2^@40 mA cm^−2^; 1.42 V; @2 mA cm^−2^ for 30 h	[48]
Co/N@CNTs@CNMF‐800	Sandwich	CNMF	KOH and PVA	0.68/63.4%	26.5 mW cm^−2^@44 mA cm^−2^; 1.40 V; @1 mA cm^−2^ for 9 h	[49]
Co–N_*x*_/C NRA	Sandwich	Ti foil	PVA	0.75/59.8%	1.27 V; @ 5 mA cm^−2^ for 8 h	[51]
FeNi@NCNTs/CC	Sandwich	CC	PVA	0.65/60.6%	7 mW cm^−2^; 1.61 V; @2 mA cm^−2^ for 36 h	[52]
Co–FeCo/N‐G	Sandwich	CC	PAM	0.65/66.1%	127 mW cm^−2^; 609 mAh g^−1^@10 mA cm^−2^; 1.42 V; @1 mA cm^−2^ for 18 h	[54]
CoNi alloy/NCNSAs/CC‐800	Sandwich	CC	KOH and PVA	0.66/65.1%	98.8 mW cm^−2^; 879 mAh g^−1^@1 mA cm^−2^; 1.41 V; @1 mA cm^−2^ for 800 min	[55]
Co SA@NCF/CNF	Sandwich	CNF	PVA	0.60/67.6%	530.17 mAh g^−1^@6.25 mA cm^−2^; 1.41 V; @6.25 mA cm^−2^ for 15 h	[56]
SCu‐ONPC	Sandwich	Graphitic carbon	KOH and PVA and ZnCl_2_	0.83/58.9%	35 mW cm^−2^@85 mA cm^−2^; @5 mA cm^−2^ for 5 h	[58]
SAFe‐SWCNT	Sandwich	SWCNT	KOH and PVA and ZnCl_2_	0.80/57.2%	33 mW cm^−2^; 1.36 V; @2 mA cm^−2^ for 10 000 s	[57]
Fe/SNCFs–NH_3_	Sandwich	CFs	PANa and KOH and Zn(CH_3_COO)_2_	1.01/51.9%	1.34 V; @ 1 mA cm^−2^ for 60 h	[60]
Co SA/NCFs	Sandwich	NCFs	PVA	0.95/53.0%	673 mAh g^−1^@6.25 mA cm^−2^; 1.42 V; @2 mA cm^−2^ for 1800 min	[59]
Ultrathin Co_3_O_4_/CC	Sandwich	CC	PVA	0.92/52.8%	542 mAh g^−1^@2 mA cm^−2^; 546 Wh kg^−1^@2 mA cm^−2^; 1.33 V; @2 mA cm^−2^ for 10 h	[64]
Co_3_O_4_–F	Sandwich	SWCNTs	PVA	0.88/54.2%	@10 mA cm^−2^ for 26 h	[62]
N_2_–Co_3_O_4_	Sandwich	CC	PVA	0.32/78.8%	32.0 mW cm^−3^@90 mA cm^−2^; 603.7 mAh g^−1^@2.5 mA cm^−2^; 1.1 V; @12.5 mA cm^−2^ for 28 h	[23b]
Co_3_O_4_–NCNT/SS	Sandwich	SS	Cellulose film	0.78/60.3%	160.7 mW cm^−2^; 652.6 mAh g^−1^@5 mA cm^−2^; 847.6 Wh kg^−1^@5 mA cm^−2^; 1.33 V; @25 mA cm^−2^ for 600 h	[44]
(Ni,Co)_3_O_4_@Ni foam	Sandwich	Ni foam	PVA	0.56/69.5%	74 mW cm^−2^@61 mA cm^−2^; 713 mAh g^−1^; @5 mA cm^−2^ for 30 h	[66]
CuCo_2_S_4_ NSs@N–CNFs	Sandwich	CNTs	PVA	0.72/64.7%	232 mW cm^−2^@280 mA cm^−2^; 896 mAh g^−1^@25 mA cm^−2^; 965.2 Wh kg^−1^@25 mA cm^−2^; 1.46 V; @5 mA cm^−2^ for 100 h	[68]
Co_9_S_8_‐NSHPCNF	Sandwich	HPCNF	PVA	0.83/58.5%	62.6 mW cm^−2^@92 mA cm^−2^; 1.338 V; @5 mA cm^−2^ for 30 h	[69]
3D N‐GQDs/NiCo_2_S_4_/CC	Sandwich	CC	KOH and PVA	1.11/50.2%	26.2 mW cm^−2^@42 mA cm^−2^; 1.406 V; @2 mA cm^−2^ for 12 h	[70]
P‐CoSe_2_/N–C FAs	Sandwich	CC	PVA	0.73/63.5%	1.30 V; @1 mA cm^−2^ for 1600 min	[63]
CoN@NC‐300	Sandwich	Ni foam	AAc and MBA and KOH	0.67/64.7%	36.68 mW cm^−2^; 680 mAh g^−1^@2 mA cm^−2^; 1.47 V; @1 mA cm^−2^ for 26.4 h	[72]
Fe–Co_4_N@N–C	Sandwich	CC	PAM and KOH and Zn(Ac)_2_	0.76/61.2%	72 mW cm^−2^@56 mA cm^−2^; 1.34 V; @4 mA cm^−2^ for 45 cycles	[73]
Co_3_O_4_/Fe_2_O_3_NAs@CNFs	Sandwich	CNFs	PVA	1.07/41.2%	150 mW cm^−2^@260 mA cm^−2^; 1.32 V; @2 mA cm^−2^ for 8 h	[74]
Co_3_O_4_@NiFe LDH	Sandwich	CC	PVA and KOH and Zn(Ac)_2_	0.96/50.3%	1.38 V; @1.3 mA cm^−2^ for 20 h	[45]
NiFeOx@VACNTs	Sandwich	CNTs	PVA	0.66/65.1%	143 mW cm^−2^@241 mA cm^−2^; 1.50 V; @1 mA cm^−2^ for 60 h	[75]
Co_9_S_8_–MoS_2_/N–CNAs@CNFs	Sandwich	CNFs	KOH and PVA	0.74/62.1%	96 mW cm^−2^@150 mA cm^−2^; @5 mA cm^−2^ for 800 min	[76]
NS@Co_3−*x*_Ni_*x*_O_4_/Co_3_O_4_	Sandwich	Ni foam	PVA	0.696/62.2%	@5 mA cm^−2^ for 200 h	[78]
Fe_3_O_4_/Fe_3_N/Eu_2_O_3_@NCG	sandwich	GO	PANa‐KOH	0.69/64.8%	92.7 mW cm^−2^@245 mA cm^−2^; 712 mAh g^−1^@2 mA cm^−2^; 854 Wh kg^−1^@2 mA cm^−2^; 1.48 V; @2 mA cm^−2^ for 460 h	[79]
NiCo_2_O_4_/NiMn LDH arrays@Ni foam	Sandwich	Ni foam	PVA and KOH and Zn(Ac)_2_	0.4/70%	1.2 V; @ 0.5 mA cm^−2^ for 30 cycles	[80]
FeP/Fe_2_O_3_@NPCA	Sandwich	GO	PVA	0.98/48.18%	40.8 mW cm^−2^@53 mA cm^−2^; 676 mAh g^−1^@5 mA cm^−2^; 517 mWh kg^−1^@5 mA cm^−2^; 1.42 V; @5 mA cm^−2^ for 500 min	[77]
NiCo_2_O_4_@N–OCNT	Cable	CNT	PVA	0.67/61.3%	3.87 mW cm^−2^; @0.5 mA cm^−2^ for 3000 s	[16a]
Co_4_N/CNW/CC	Cable	CC	PVA	0.92/54.7%	1.35 V; @1.0 mA cm^−2^ for 12 h	[12a]
Co3O_4_/N‐rGO	Cable	GO	PVA	0.80/60.0%	550 mAh g^−1^@6 mA cm^−2^; 649 Wh kg^−1^@6 mA cm^−2^; 1.31 V; @3.0 mA cm^−2^ for 25 h	[12b]
Co/Co–N–C	Cable	Carbon felts	PVA	0.72/62.7%	1.41 V; @5.0 mA cm^−2^ for 10 h	[141]

### 3.1 Metal‐Free Carbon Materials

High electronic conductivity, large specific surface area, and diverse morphologies are the unique characteristics of metal‐free carbon materials used in self‐supported bifunctional air electrodes for FSZABs. However, the ORR of these materials generally proceeds in a two‐electron pathway (Equation (5) and (6)), leading to the aforementioned low energy efficiency and stability. At the same time, carbon materials possess poor OER activity and tend to oxidize and corrode at high OER potentials, reducing the stability and energy conversion efficiency of ZABs.^[^ ^33^ ^]^

**Figure 4:**
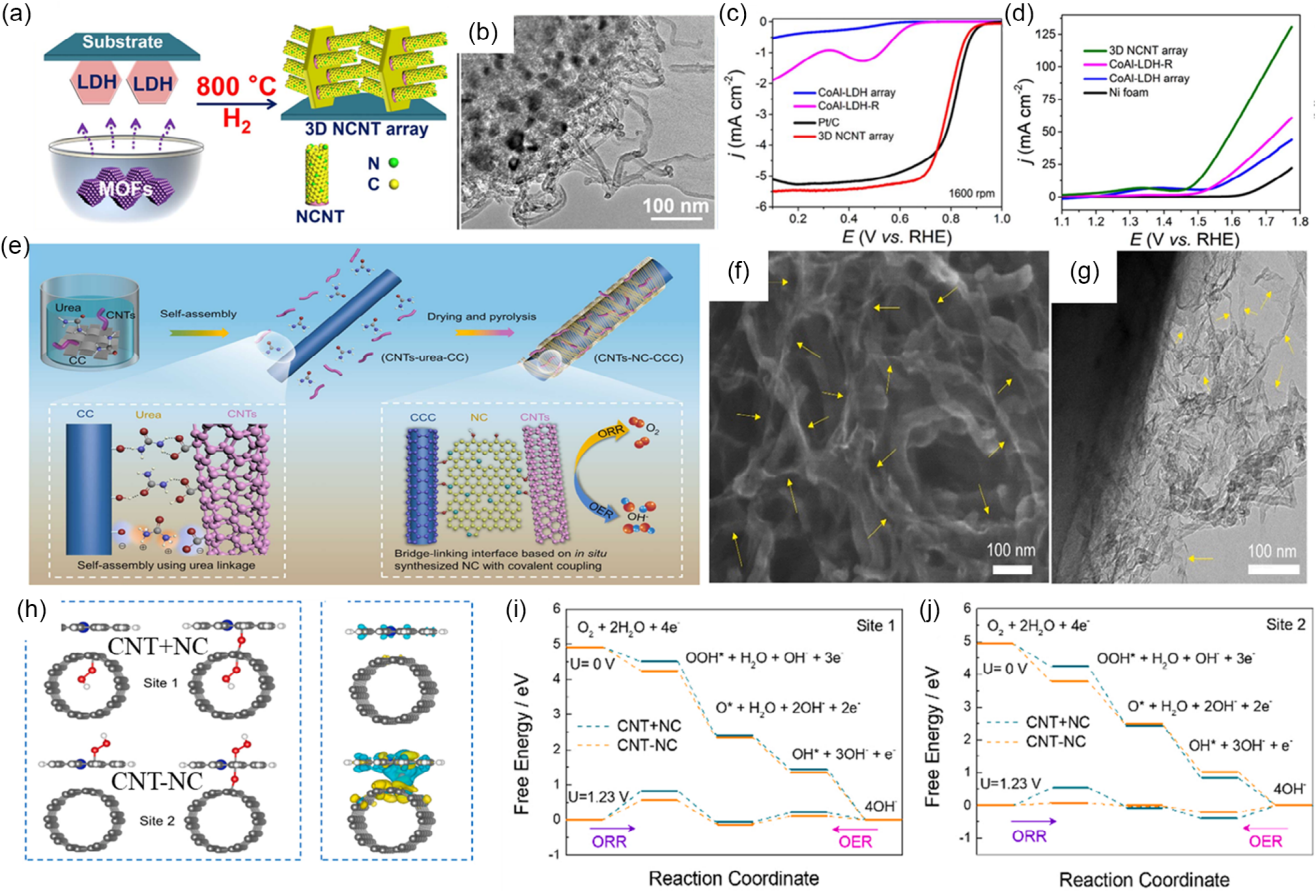
a) Schematic illustration of the synthesis of 3D NCNT arrays by a facile‐directed growth process and b–d) their corresponding TEM image (b) and ORR and OER performance (c,d), respectively. a–d) Reproduced with permission.^[^ ^36^ ^]^ Copyright 2017, Elsevier. e) Schematic illustration for synthesis of CNTs–NC–CCC and their corresponding f,g) SEM and TEM images, h) spin–charge density distribution of CNT + NC and CNT–NC, and i,j) free energy diagrams of CNT + NC and CNT–NC on site 1 (i) and site 2 (j) for ORR and OER, respectively. e–j) Reproduced with permission.^[^ ^39^ ^]^ Copyright 2022, Elsevier.

However, studies have reported that doping heteroatoms (N, S, O, B, and P) can break the C—C bonds and promote electron transfer, thus optimizing active sites’ adsorption–desorption characteristics.^[^ ^34^ ^]^ Heteroatom doping on carbon substrates can also lessen their oxidative decomposition during the OER (charging) process.^[^ ^35^ ^]^ A few recent studies have successfully developed self‐supported bifunctional air electrodes using these heteroatom‐doped carbon materials (metal‐free) that can concurrently overcome the setbacks of ORR and OER,[^34^, ^35^] including not only single but also binary or ternary heteroatom dopants. Li et al.^[^ ^36^ ^]^ adopted a directed growth approach to developing 3D nitrogen‐doped carbon nanotube (NCNT) arrays/Ni foam as self‐supported bifunctional air electrodes, where their metal–organic frameworks (MOFs) serve as carbon and nitrogen source (**Figure** **4**a). N‐doping with strong electron affinity can increase the positive charge density of adjacent carbon atoms, enabling the O_2_ adsorption and O═O bond breaking, thus speeding up the O_2_ reduction rate in the aforementioned four‐electron pathway (Equation (7)–(10)).^[^ ^37^, ^38^ ^]^ As a result, with its uniform N‐doping, unique hierarchical nanoarray structure (Figure 4b), and decreased charge transfer resistance, the developed 3D NCNT array could achieve an *E*_1/2_ of 0.81 V (Figure 4c) and an *η* of 0.27 V (Figure 4d).

Similarly, Zheng et al.^[^ ^39^ ^]^ adopted the self‐assembly and covalent coupling method and successfully prepared a CNT on carbonized carbon cloth (CCC) linked with N‐doped nanocarbons (NC) electrode (CNTs–NC–CCC) (Figure 4e). They used the urea molecules to link CNTs and CC to achieve self‐assembly. Then, during the carbonization process, the spider‐web‐like NC was formed and acted as the conductive agent and binder to intertwine with CNTs, connecting the catalysts and substrates firmly based on covalent linkage and thus producing a 3D conductive and hierarchical porous network (Figure 4f,g). Next, they performed the density functional theory (DFT) calculations to reveal the reaction mechanism for this intertwined interface of CNTs–NC–CCC. With such interface bonding of CNT–NC, they managed to modulate the local charge density distribution and enhanced electron transfer, thus forming noticeable charge density differences compared to that of CNT + CC (Figure 4h). The CNT–NC also exhibited a smaller energy barrier for ORR and OER than that of CNT + NC at sites 1 and 2 (Figure 4i,j), thus acquiring a low Δ*E* of 0.78 V. In other words, the covalent coupling can develop a strong and stable interfacial connection between catalyst and substrate, increasing active sites and stabilizing the structure during the operation of ZABs. In short, we can consider interface engineering an efficient strategy to improve electrocatalysts’ electrochemical activity and durability.

**Figure 5:**
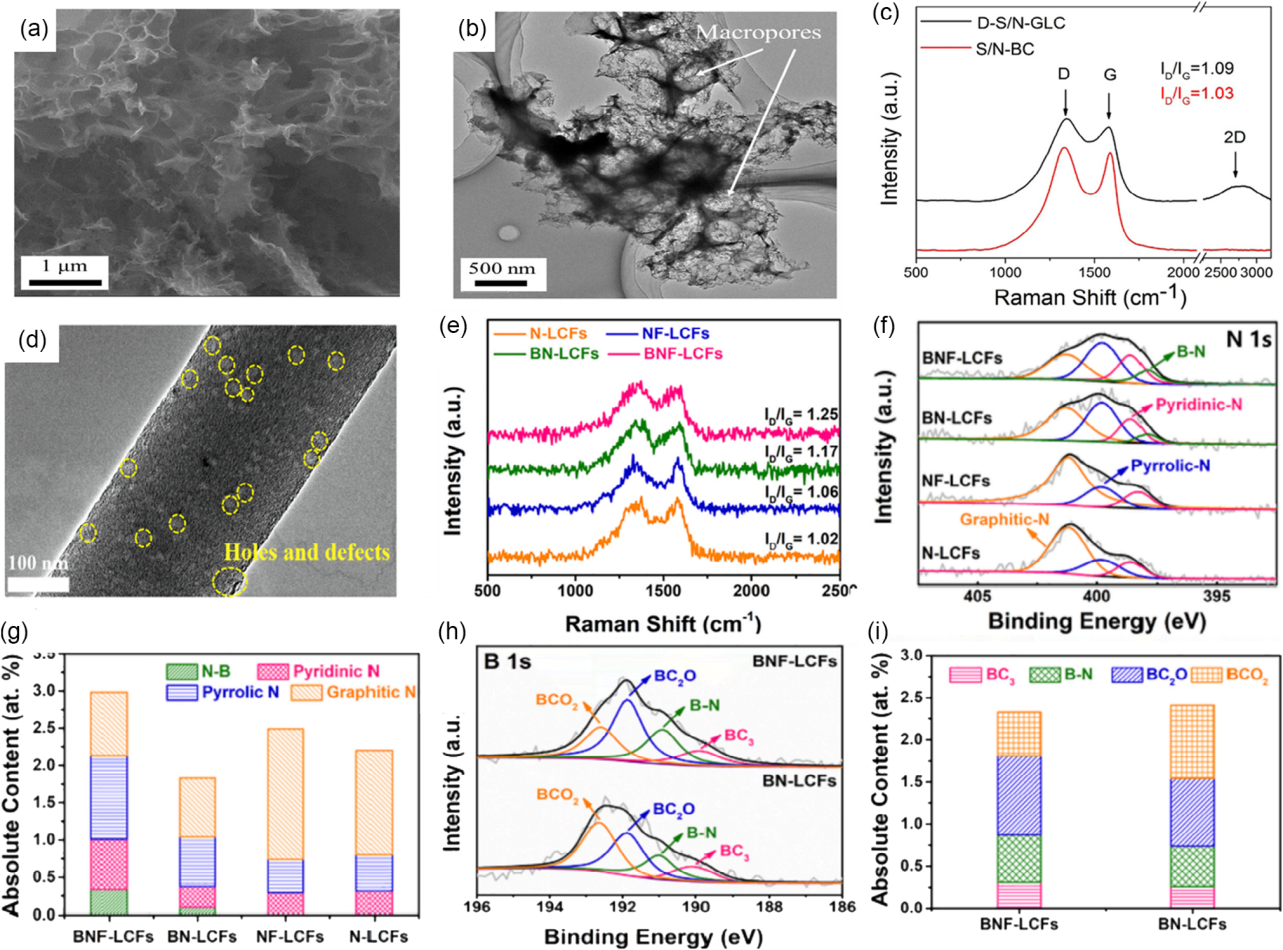
a,b) TEM images and c) Raman spectra of D‐S/N‐GLC. a–c) Reproduced with permission.^[^ ^41^ ^]^ Copyright 2017, American Chemical Society. d) TEM image of BNF‐LCFs. e) Raman spectra of catalysts. f) N 1*s* spectra of catalysts. g) The absolute content of various N species in catalysts. h) B 1*s* spectra of catalysts. i) The absolute content of various N species in catalysts. d–i) Reproduced with permission.^[^ ^42^ ^]^ Copyright 2022, Elsevier.

Codoping with different heteroatoms (other than N) can control the partial electronic structure and carbon polarity effectively, thus enhancing further the electrochemical activity and durability of the carbon materials.^[^ ^40^ ^]^ Zhang et al.^[^ ^41^ ^]^ successfully used defect‐enriched porous graphene‐like carbon nanomaterial with N, S atoms codoping (D‐S/N‐GLC) as an air electrode for the FSZAB (**Figure** **5**a–c), which showed a peak power density of 81 mW cm^−2^. Likewise, Wang et al.^[^ ^42^ ^]^ motivated by this codoping strategy, successfully developed B, N, and F tridoped lignin‐based interconnected carbon nanofibers (BNF‐LCFs) electrodes via electrospinning and pyrolysis. They coadded ammonium fluoride and zinc borate to the BNF‐LCFs to increase their porosity, holes, and defective structures (Figure 5d,e). In addition, their X‐ray photoelectron spectroscopy (XPS) spectra and the calculated bar charts revealed that adding B and F could influence the concentration and species of N functional groups (Figure 5f–i), thus optimizing the carbon matrix's electronic structures and electrochemical properties. As a result, their samples exhibited excellent bifunctional activity toward ORR and OER with a small Δ*E* of 0.73 V. Moreover, the FSZAB assembled with BNF‐LCFs could deliver an OCV of 1.36 V and display impressive cycling stability for 13 h.

### 3.2 Transition Metals and Compounds/Conductive Substrates

Although the aforementioned heteroatom‐doped metal‐free carbon materials can act as self‐supported air electrodes to relieve carbon decomposition during the OER (charging), this oxidative decomposition cannot be avoided entirely, notably during a long‐term cycling test. This decomposition will cause the electrolyte turn yellow and severely reduce battery performance.^[^ ^43^ ^]^ Hence, transition metals and compounds have attracted much attention for developing stable catalysts by directly using them on the conductive carbon surface to replace metal‐free carbon electrodes. These transition metals and compounds can be metals, alloys, metal oxides, sulfides, nitrides, and phosphides. Their multiple oxidation states and active sites provide abundant catalytic sites for ORR and OER when constructed on the carbon surface or interacting with the conductive substrate.[^16^, ^44^, ^45^] They can also mitigate electrochemical corrosion and carbon decomposition. In short, metal materials can also be employed as substrates for air electrodes to improve long‐term cycling stability given their excellent stability.

#### 3.2.1 Transition Metals

**Figure 6:**
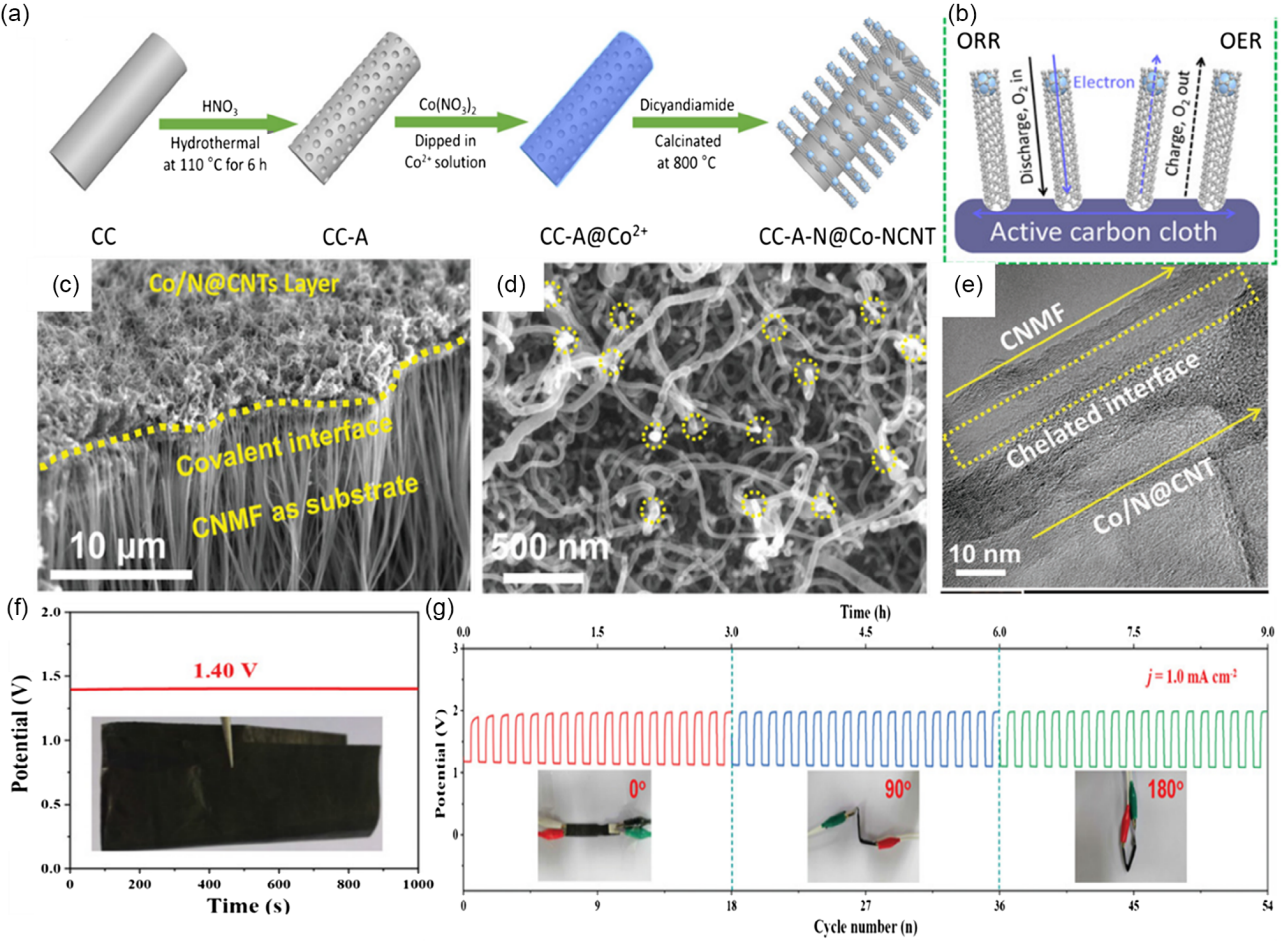
a,b) Schematic illustration of the synthesis (a) and ORR and OER mechanisms (b) of the CC‐A‐N@Co–NCNT. a,b) Reproduced with permission.^[^ ^48^ ^]^ Copyright 2020, Wiley‐VCH. c,d) SEM and e) HRTEM images of Co/N@CNTs@CNMF‐800. f) OCV test, and g) charge–discharge profiles of ZABs at different angles. c–g) Reproduced with permission.^[^ ^49^ ^]^ Copyright 2020, Wiley‐VCH.

Conductive substrates can help to disperse active sites effectively and increase the electrochemical activity, thus preventing the agglomeration and corrosion of metal nanoparticles.^[^ ^46^, ^47^ ^]^ Therefore, constructing transition metal nanoparticles on conductive substrates and N‐doping to form self‐supported bifunctional air electrodes can be an effective strategy to enhance battery performance. For instance, Lu et al.^[^ ^48^ ^]^ successfully introduced cobalt (Co), N‐codoped CNT arrays on carbon fiber cloth (CC‐A‐N@Co–NCNT) through a two‐step in situ growth method (**Figure** **6**a),^[^ ^48^ ^]^ producing an air electrode with the best oxygen diffusion path and the shortest electron‐transport path (Figure 6b), thus delivering superior flexibility and stability for ZABs. Similarly, Liu et al.^[^ ^49^ ^]^ developed a 3D hierarchical porous electrode by combining in situ growth and deposition methods, producing Co/N@CNTs@CNMF‐based FSZABs, where CNMF represents carbon nanotube microfilm. Their scanning electron microscopy (SEM) and high‐resolution transmission electron microscopy (HRTEM) images showed Co nanoparticles captured in bamboo‐like N‐doped CNTs on the CNMF surface (Figure 6c–e). As a result, their ZAB could deliver a high OCV of 1.40 V (Figure 6f), a peak power density of 26.5 mW cm^−2^, and a stable charge–discharge cycling curve at different bending angles (Figure 6g).

Recently, zeolitic‐imidazolate framework (ZIF) materials become popular among transition metals and compounds for developing bifunctional catalysts. ZIF, which consists of metal cations and imidazole ligands, displays ultrahigh specific surface area, high porosity, and structural flexibility advantages.^[^ ^50^ ^]^ ZIF‐derived porous carbon frameworks with unique morphologies of metal nanoparticles and/or N atom doping via the pyrolysis process play a vital role in transition metals and compounds. For example, Amiinu et al.^[^ ^51^ ^]^ used 3D ZIF nanocrystals to obtain Ti foil for preparing a novel Co–N_*x*_/C. This bifunctional catalyst could demonstrate a low Δ*E* of 0.65 V for ORR and OER, a high OCV of 1.42 V, a high voltaic efficiency of 65.7%, and an excellent cycling stability for FSZABs. Such outstanding electrochemical activity can be ascribed to the presence of numerous active sites on the nanorods, increased surface area, and synergistic effect of the abundant Co–N coupling centers, which were confirmed by their XPS and DFT analysis results.

**Figure 7:**
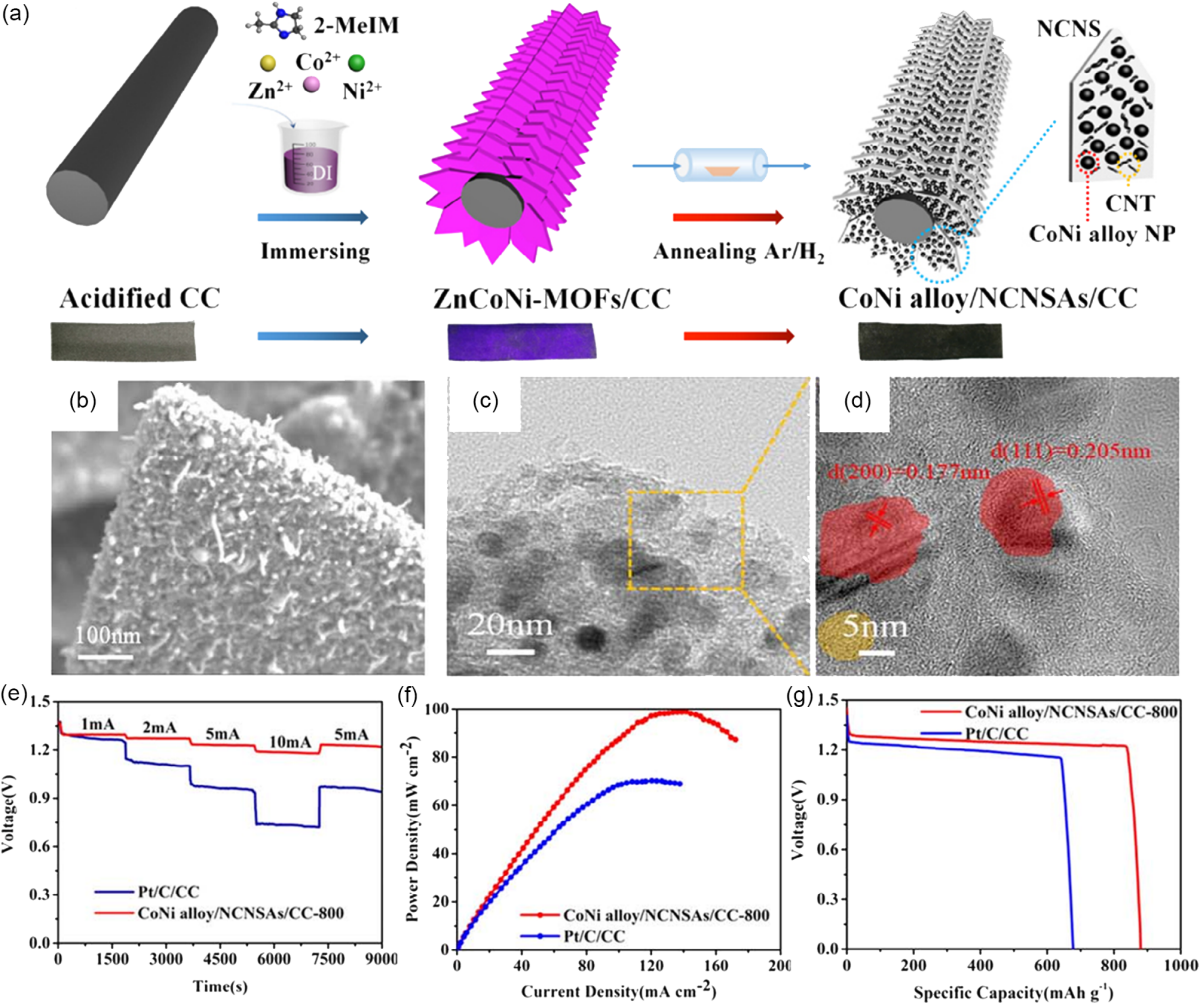
CoNi alloy and CNT decorated N‐doped carbon nanosheet arrays on carbon cloth. a) Schematic illustration of the synthesis process. b) SEM images. c,d) HRTEM images. e) Galvanostatic discharge profiles at different current densities. f) Power–current density profiles. g) Voltage–capacity profiles. a–g) Reproduced with permission.^[^ ^55^ ^]^ Copyright 2020, IOP Publishing Ltd.

Similarly, constructing bimetallic alloys (Fe, Co, and Ni) on conductive substrates with N‐doping can enhance the air electrodes’ ORR and OER electrochemical activities through the electronic interactions between different metals.^[^ ^52^, ^53^ ^]^ For instance, Jin et al.^[^ ^54^ ^]^ managed to encapsulate Co and FeCo nanowire arrays on N‐doped carbon cloth (Co–FeCo/N‐G‐CC) as air electrodes for FSZABs. The superior electrochemical activity can be ascribed to the etching effect and plasma that enhanced the porosity and exposed more active sites for the Co–FeCo/N‐G‐CC. Typically, the alloys can also be derived from MOFs during pyrolysis. Zhang et al.^[^ ^55^ ^]^ successfully fabricated a bifunctional catalyst CoNi alloy/NCNSAs/CC with CoNi alloy and CNT decorated N‐doped carbon nanosheet arrays on CC (NCNSAs/CC) (**Figure** **7**a). The surface morphology revealed uniform impaction of CoNi nanoparticles and CNTs in the NCNS on CC (Figure 7b–d), thus giving enhanced bifunctional performance toward ORR and OER with a marginal potential difference of 0.68 V. This enhancement can be ascribed to the 3D hierarchical nanostructure, evenly dispersed active sites, and high graphitization degree. Furthermore, the FSZABs based on CoNi alloy/NCNSAs/CC as a self‐supported air electrode demonstrated superior durability (Figure 7e), and higher powder density (98.8 mW cm^−2^) (Figure 7f) and capacity (879 mAh h^−1^) than those of Pt/C/CC (Figure 7g).

**Figure 8:**
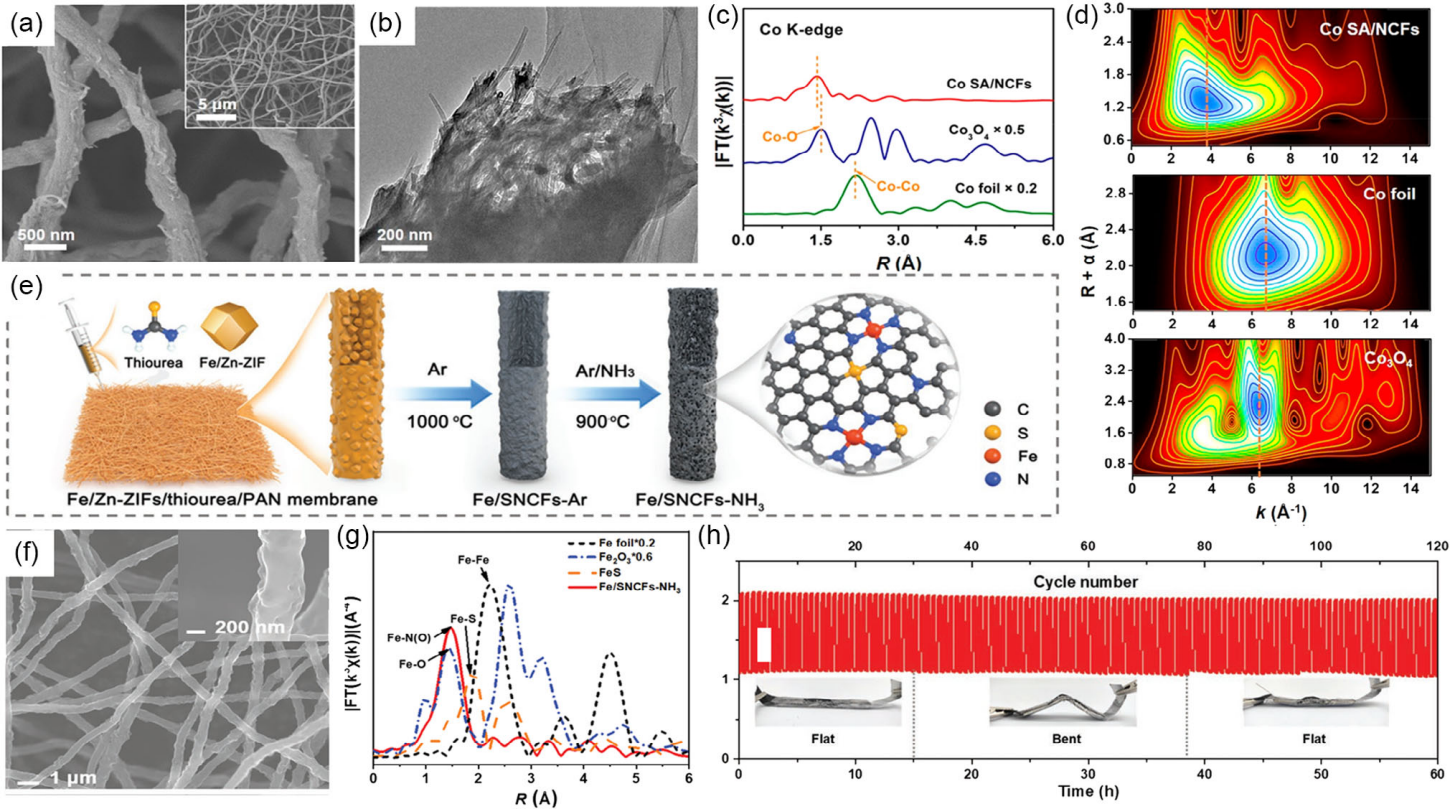
a) SEM and b) TEM images of Co SA/NCFs. c) Fourier transforms and d) WT analysis of EXAFS spectra for samples. a–d) Reproduced with permission.^[^ ^59^ ^]^ Copyright 2022, American Chemical Society. e) Schematic illustration of the synthesis and f) SEM image of Fe/SNCFs–NH_3_. g) Fourier transforms of EXAFS spectra of samples. h) Charge–discharge profiles of catalysts. e–h) Reproduced with permission.^[^ ^60^ ^]^ Copyright 2022, Wiley‐VCH.

On the other hand, combining atomic dispersion of metal atoms with heteroatom‐doped conductive carbon substrates can be another effective strategy to optimize the utilization efficiency and electrocatalytic performance of FSZABs.^[^ ^56^, ^57^ ^]^ For instance, Wang et al.^[^ ^58^ ^]^ pyrolyzed Cu^2+^‐saturated brinjal slice to obtain a single Cu atom anchored O,N‐doped porous carbon (SCu‐ONPC) air electrode. Their DFT analysis revealed that the Cu–N_3_O species contributed to increased intrinsic activity both for ORR and OER. Interestingly, M–N_*x*_ species anchoring in conductive carbon substrates often act as active sites for ORR and OER. They used an electrospinning method to disperse Co atoms and construct CNT‐linked NCFs in Co SA/NCFs catalysts. Their SEM, transmission electron microscopy (TEM), X‐ray absorption near edge spectroscopy (XANES), and extended X‐ray absorption fine structure (EXAFS) analysis revealed the formation of randomly arranged 3D network structure (**Figure** **8**a,b) and atomic‐level dispersion of Co atoms in the form of Co—N bonds (Figure 8c,d). These observations support the excellent electrochemical performance.^[^ ^59^ ^]^

Similarly, Yang et al.^[^ ^60^ ^]^ combined electrospinning and pyrolysis to prepare a self‐supported bifunctional air electrode by dispersing Fe–N_4_/C atomically on the carbon fiber membrane (Fe/SNCFs–NH_3_) (Figure 8e). The SEM image showed the presence of abundant interconnected macropores formed by the intertwined carbon fibers (Figure 8f), which facilitate gas transport, electrolyte infiltration, and electron transfer. Moreover, the EXAFS curves verified that Fe in Fe/SNCFs–NH_3_ is atomically dispersed and coordinated with N/O (Figure 8g). Notably, doping of S atoms plays a vital role in modulating reaction barriers to improve ORR and OER processes. Therefore, this bifunctional catalyst displayed the excellent activity for ORR, OER, and solid‐state ZABs (Figure 8h).

#### 3.2.2 Transition Metal Compounds/Conductive Substrates

Recently, transition metal‐based oxides, sulfides, nitrides, phosphides, and selenides have been extensively explored as bifunctional oxygen electrocatalysts. However, the poor conductivity and low dispersion of these compounds in the powder form still hinder the catalytic improvement of ZABs. Thus, transition metal compounds are usually deposited onto conductive substrates surface to overcome the aforementioned deficiencies. In particular, Co‐, Fe‐, and Ni‐based single metal oxides, spinel oxides, and perovskite oxides have attracted much attention given their low cost, simple preparation, environmental compatibility, and natural abundance.^[^ ^61^ ^]^ Studies showed that coating the transition oxides onto conductive substrates to obtain self‐supported air electrodes with the novel nanostructure could increase the specific surface area, conductivity, durability, and electron transfer rate, and shorten the ion diffusion length.^[^ ^62^ ^]^

Co‐based transition metal oxides, especially Co‐based spinel‐type oxides, show excellent electrocatalytic activity given their excellent stability, multiple oxidation states of Co ions, and unique structure. Spinel Co_3_O_4_ has two types of Co ions, i.e., Co^3+^ ions at octahedral sites that promote the ORR and Co^2+^ ions at tetrahedral positions that favor the OER process. However, their limited active sites, low specific surface area, and poor conductivity have severely limited their electrocatalytic activity. Hence, constructing the spinel Co_3_O_4_ on carbon materials or using the conductive substrates to construct self‐supported air electrodes can be an efficient pathway for FSZABs.^[^ ^62^, ^63^ ^]^

**Figure 9:**
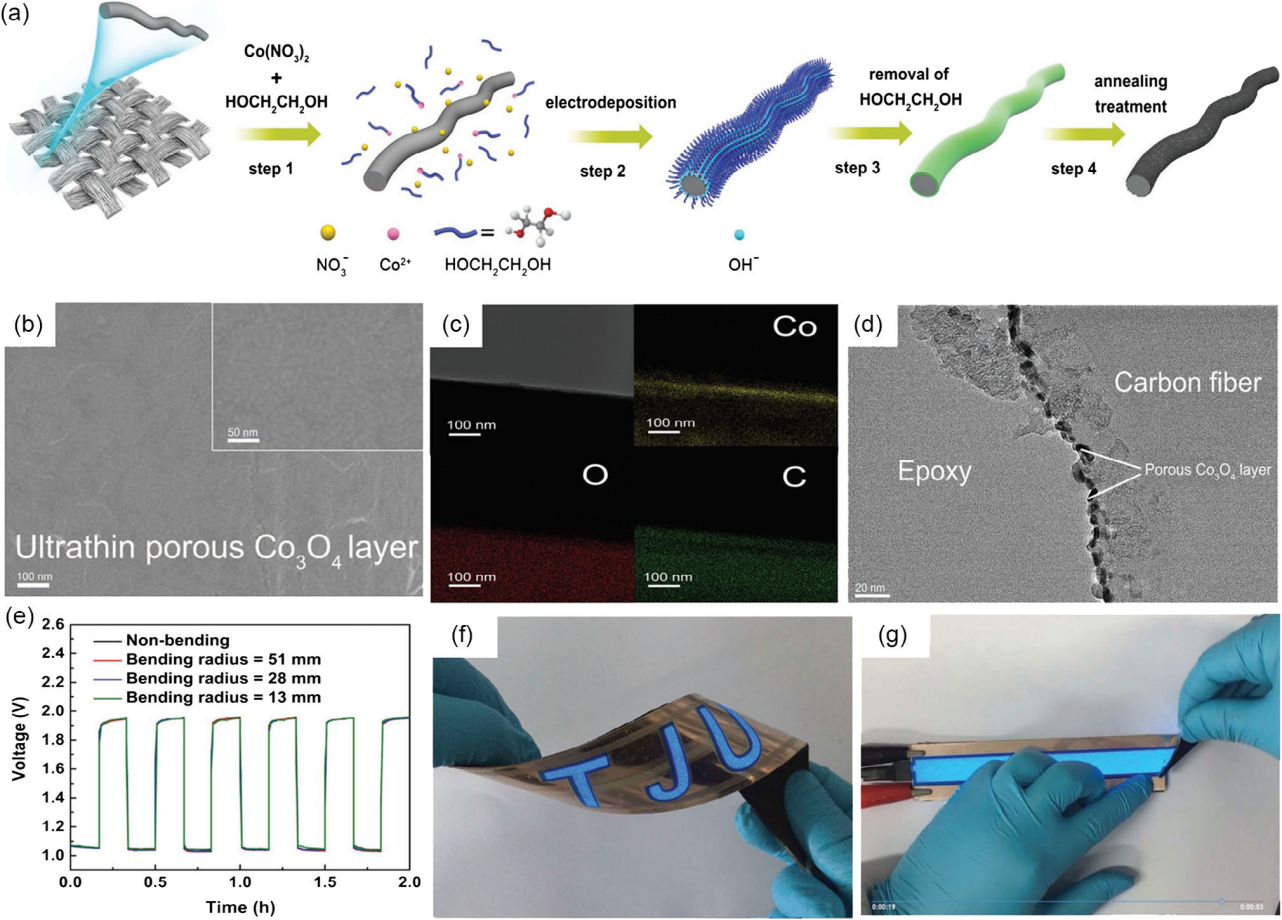
a) Schematic illustration of the ultrathin Co_3_O_4_/CC synthesis process. b) SEM and c,d) TEM images and their corresponding elemental mapping. e) Galvanostatic charge–discharge test results of the FSZABs in the panel under various bending radii of 51, 28, and 13 mm. f,g) Photographs of a working integrated device during the cutting process. a–g) Reproduced with permission.^[^ ^64^ ^]^ Copyright 2017, Wiley‐VCH.

Chen et al.^[^ ^64^ ^]^ grew the ultrathin Co_3_O_4_ layer on the carbon fibers horizontally and uniformly by electrodeposition followed by annealing treatment (**Figure** **9**a). The surface morphology showed the presence of mesopores in the Co_3_O_4_ layer and ultrathin Co_3_O_4_ layer (5 nm) (Figure 9b–d), resulting in an optimized electrical contact area and strong adhesion on the conductive support. As a result, the FSZAB exhibited excellent mechanical stability and safety under severe conditions (Figure 9e–g). Yu et al.^[23b]^ managed to fabricate N‐doped Co_3_O_4_ nanowires on CC through hydrothermal and sequential annealing, giving a high 98.1 mAh cm^−3^ volumetric capacity and flexibility with its air electrode for the FSZABs. Their results confirmed that N‐doping in Co_3_O_4_ could also enhance the electronic conductivity, O_2_ adsorption strength, and reaction kinetics, thus improving the overall electrochemical activity. These excellent outcomes highlight its practical suitability for flexible and wearable electronics application.

Another common strategy to develop self‐supported air electrodes for FSZABs is through in situ synthesis of array structures such as hydrothermal, chemical, or electrochemical with subsequent pyrolysis. These array structures with orientated growth and high surface area are constructed on the conductive carbon surface or metal substrates, improving the close contact of the components in the air electrode and promoting oxygen and electrolyte diffusion to reach the active catalytic site efficiently, consequently accelerating the oxygen reaction process. At the same time, direct growth of bifunctional catalysts on flexible substrates can benefit fast electron transfer, good conductivity, and structure stability, enabling high‐performance ZABs.^[^ ^65^ ^]^

**Figure 10:**
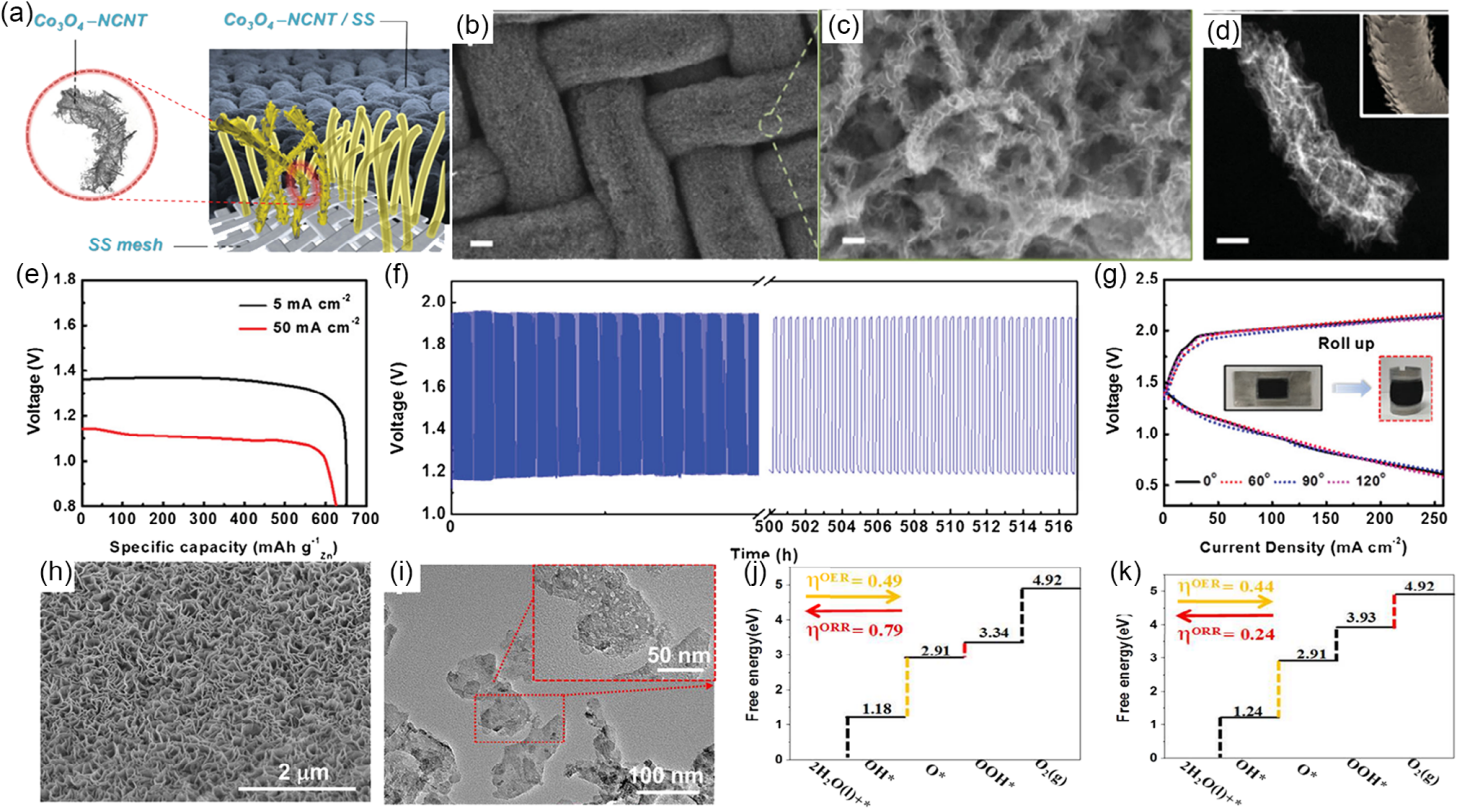
a) A schematic illustration of a hair‐like array of Co_3_O_4_–NCNT/SS air electrode. b,c) SEM and d) TEM images of Co_3_O_4_–NCNT/SS. e) Specific capacity, f) charge–discharge, and g) polarization profiles at different bending angles of Co_3_O_4_–NCNT/SS‐based ZAB. a–g) Reproduced with permission.^[^ ^44^ ^]^ Copyright 2016, Wiley‐VCH. h) SEM and i) TEM images of (Ni, Co)_3_O_4_@Ni foam. j) Free energy diagrams of OER and ORR on the Co site surface of Co_3_O_4_ (001) at the *U* = 0 V. k) Free energy diagrams of OER and ORR on the Co site surface of Ni‐doped Co_3_O_4_ (001) at the *U* = 0 V. h–k) Reproduced with permission.^[^ ^66^ ^]^ Copyright 2020, Elsevier.

Therefore, Fu et al.^[^ ^44^ ^]^ embedded Co_3_O_4_ nanoparticles in NCNT arrays on the stainless steel (SS) mesh surface, developing a unique morphology structure similar to human hair arrays (**Figure** **10**a). Benefiting from this hair‐like structure (Figure 10b–d) and synergistic effects between Co_3_O_4_ and NCNT, the Co_3_O_4_–NCNT arrays can be coupled with SS mesh without sacrificing interfacial contact. Hence, these arrays enabled high electrical conductivity, boosting electrocatalytic active sites and preventing Co_3_O_4_–NCNT from restacking and detaching. As a result, the Co_3_O_4_–NCNT/SS‐based FSZAB could yield a high specific capacity of 652.6 and 632.3 mAh g^−1^ at 5 and 50 mA cm^−2^, respectively (Figure 10e), excellent rechargeable stability (Figure 10f), and robust mechanical integrity (Figure 10g).

Moreover, introducing metal cations as in the case of Co‐based spinel oxides with nanostructure can also enrich the active sites. They can impart new electrochemical activities due to the electron exchange interaction, thus enhancing the electrochemical activity of catalysts.^[16a]^ Besides, the Ni atom radius of 149 pm is close to that of Co of 152 pm, making it easy to access the crystal cell of Co_3_O_4_ and weaken the lattice alkalinity. For example, Xu et al.^[^ ^66^ ^]^ deposited (Ni,Co)_3_O_4_ oxide onto Ni foam to construct self‐supported air electrodes for FSZABs. Their SEM images revealed consistent growth of (Ni,Co)_3_O_4_ nanosheet arrays in Ni form with many pores in (Ni,Co)_3_O_4_, allowing the oxygen and electrolyte access during the electrochemical reaction process (Figure 10h,i). Then, their DFT calculation revealed the ORR and OER mechanisms (Figure 10j,k). As for the OER, the rich defect sites in (Ni, Co)_3_O_4_ nanosheet arrays accelerated the adsorption of reactants. During the ORR, the Ni‐doping in Co_3_O_4_ lowered the surface activity toward O, OH, and OOH species, further keeping the OOH molecular state without dissociation on the surface, thus improving the ORR activity. As a result, this self‐supported electrode yielded a lower charge–discharge gap of 0.56 V, a large specific volumetric energy capacity of 2268 mW h cm^−3^, as well as a high specific energy capacity of 686 mW h g^−1^.

Recent studies have shown that self‐supported bifunctional air electrodes were successfully developed using transition metal chalcogenides/conductive substrates.^[^ ^67^, ^68^ ^]^ This can be attributed to their suitable binding energy to intermediates reaction, favorable electrical conductivity, and corrosion resistance. Peng et al.^[^ ^69^ ^]^ embedded Co_9_S_8_ nanoparticles in N/S dual‐doped hollow and porous carbon nanofibers through electrospinning followed by reoxidation and carbonization treatment. Due to the unique structure, dual doping of N and S atoms, enhanced electrical conductivity, and high specific surface area, the as‐prepared Co_9_S_8_‐NSHPCNF yielded an *E*_1/2_ of 0.82 V for ORR and *η* of 0.35 V for OER, outperforming the precious metal‐based catalysts. Liu et al.^[^ ^70^ ^]^ designed a 3D NiCo_2_S_4_ nanoarrays with defect‐enriched N‐doped GQDs (N‐GQDs) on CC as a bifunctional air electrode. Their DFT calculations revealed that the stronger the OOH* dissociation adsorption at the interface between N‐GQDs and NiCo_2_S_4_, the lower the overpotential between ORR and OER. Hence, given its unique morphologies and high conductivity, the CoSe_2_ catalyst plays a vital role in transition metal chalcogenides.

**Figure 11:**
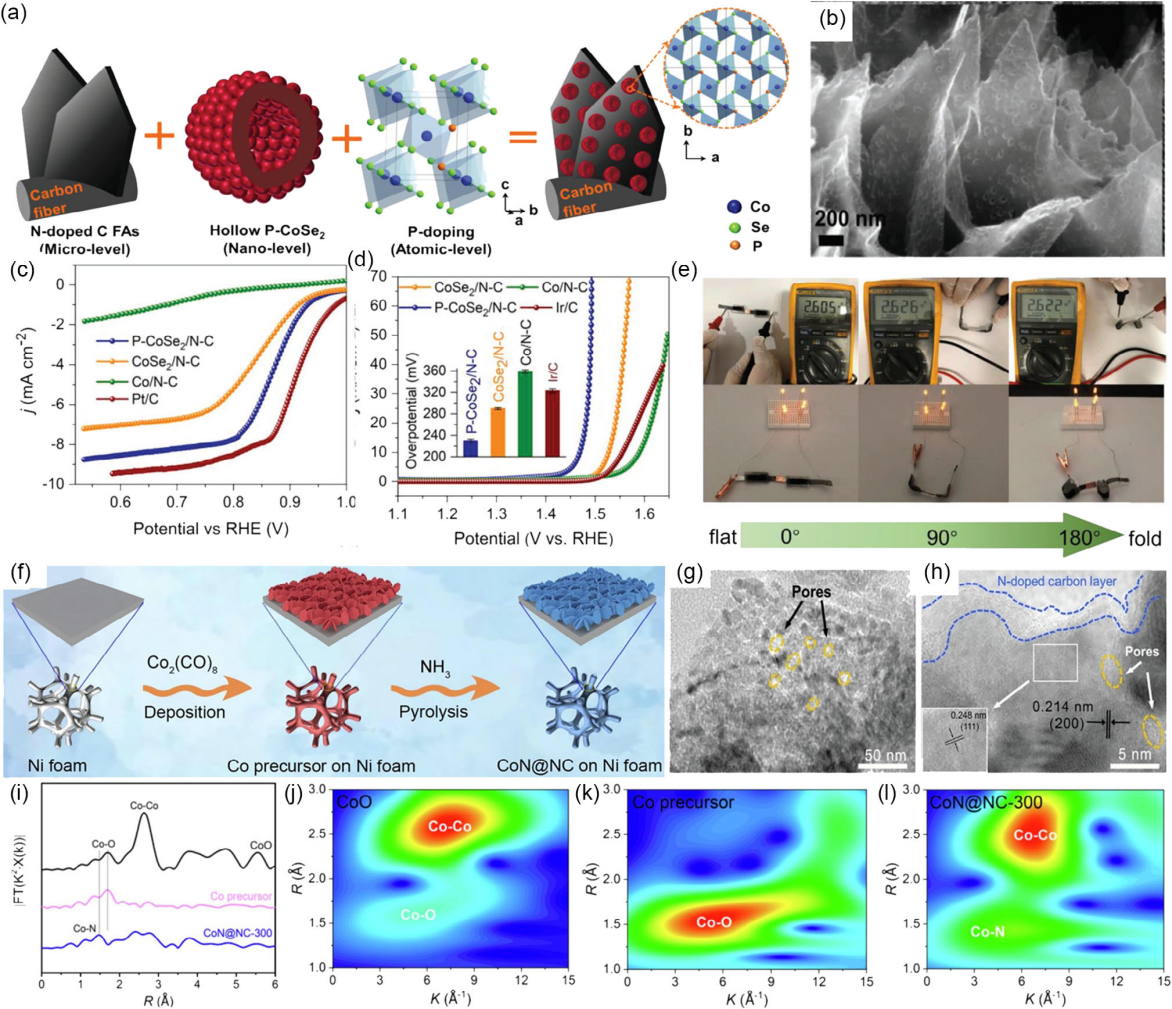
a) Schematic illustration of the synthesis process of P‐CoSe_2_/N–C arrays. b) SEM image of P‐CoSe_2_/N–C. c) ORR and d) OER profiles and e) digital images of the OCVs and LEDs powered by P‐CoSe_2_/N–C‐based ZAB at different bending angles. a–e) Reproduced with permission.^[^ ^63^ ^]^ Copyright 2018, Wiley‐VCH. f) Schematic illustration of the synthesis process of CoN@NC. g) TEM and h) HRTEM images of CoN@NC‐300. i) Fourier transform extended XANES spectra and j–l) wavelet transforms of CoO (j), Co precursor (k), and CoN@NC–300 (l). f–l) Reproduced with permission.^[^ ^72^ ^]^ Copyright 2022, Elsevier.

To obtain self‐supported bifunctional air electrodes for FSZABs, Zhang et al.^[^ ^63^ ^]^ prepared hierarchical flake arrays of N‐doped carbon flakes embedded with P‐doped CoSe_2_ on CC (**Figure** **11**a). Such hierarchical flake arrays in P‐CoSe_2_/N–C (Figure 11b) displayed an *E*_1/2_ of 0.87 V for ORR (Figure 11c) and *η* of 0.23 V for OER (Figure 11d). In addition, the FSZAB yielded a high OCV of 1.30 V and good mechanical flexibility (Figure 11e). These much‐enhanced electrocatalytic performances can be ascribed to the synergistic interaction of the multilevel controls in the structure as well as the optimized electronic structure resulted by P‐doping. With the exception of metal chalcogenides, combining transition metal nitrides with conductive substrates has also led to the outstanding activity of ORR and OER. Their excellent electron transfer ability and abundant elemental oxidation states played great significance in regulating the adsorption energy and conductivity.

Except for the metal chalcogenides, the combination of transition metal nitrides with conductive substrates has also been proved with superior ORR and OER activity because of their excellent electron transfer ability and abundant elemental valence states, which is of great significance for regulating the adsorption energy and conductivity.^[^ ^71^ ^]^ Song et al.^[^ ^72^ ^]^ successfully grown hierarchically porous cobalt nitride hybrid nanosheets on Ni foam (CoN@NC) via a novel vaporization–nitridation synthesis strategy (Figure 11f). The TEM and HRTEM images (Figure 11g,h) verified the embedding of CoN nanoparticles in amorphous NC layer with abundant pores. The extended XAFS spectra in Figure 11i demonstrated the transformation of Co—O bond into Co—N in CoN@NC‐300 after nitridation treatment, which is consistent with the results of the wavelet transform analysis (Figure 11j–l). With this 3D hierarchical porous architecture and strong coupling effect between CoN and NC, the flexible catalyst yielded an OCV of 1.47 V, a power density of 36.68 mW cm^−2^, a specific capacity of 680 mAh g_Zn_^−1^, and good cycling stability. Similarly, the codoped Fe–Co_4_N@N–C nanosheet array derived from MOFs could also yield high electrochemical activity for ORR (*E*_1/2_ of 0.83 V) and OER (*E*_10_ of 1.62 V).^[^ ^73^ ^]^ The abundant pyridinic‐N–M active sites for ORR and the enriched Co^3+^ sites for OER in Fe–Co_4_N@N–C boosted the reaction kinetics. As a result, the FSZAB employing such air electrodes could yield a high volumetric power density of 72 mW cm^−3^ and excellent cycling durability under different bending states.

### 3.3 Other Catalysts

Combining the advantages of two or more materials by structural design on the substrate's surface can concurrently improve air electrodes’ electrochemical activity and stability. Several catalysts, including metal oxides, metal chalcogenides, and metal hydroxides, have been used to develop composites on conductive substrates.^[^ ^45^, ^74^, ^75^, ^76^, ^77^ ^]^ Among them, combining two types of transition metal oxides to develop self‐supported air electrodes has been proven promising.^[^ ^74^ ^]^ For example, Wu et al.^[^ ^78^ ^]^ synthesized a self‐supported sandwich structured electrode by pressing two electrodes (NF@Co_3−*x*_Ni_*x*_O_4_ and SS@Co_3_O_4_) together. Their XAFS measurement revealed that the doping of Ni in Co_3_O_4_ could change the local geometry and electronic structure of Co_3_O_4_. With this sandwich structure, the electrode yielded a high energy efficiency of 62.2% at a 5 mA cm^−2^ current density for FSZABs.

**Figure 12:**
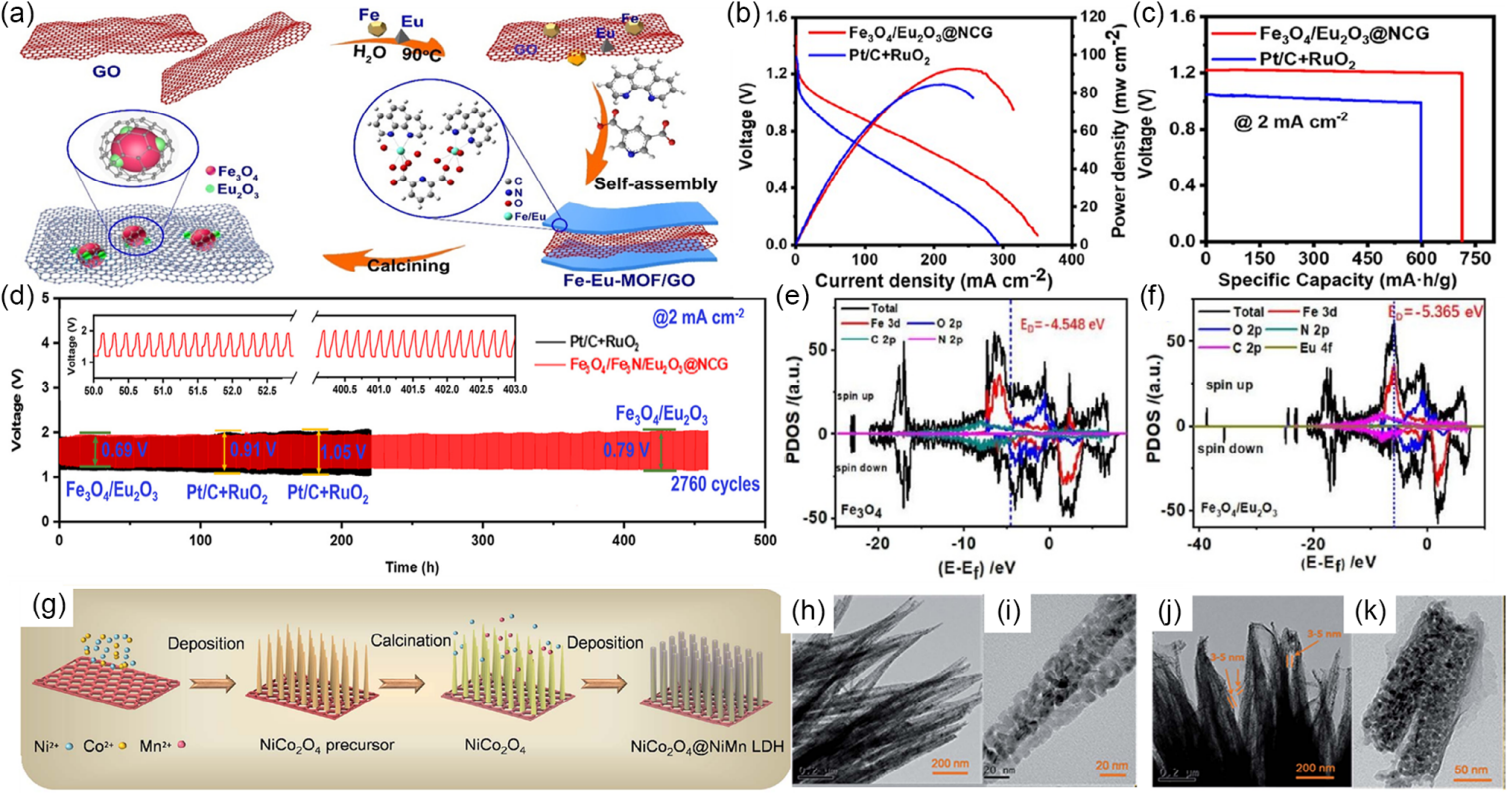
a) Schematic illustration of the synthesis process of Fe_3_O_4_/Eu_2_O_3_@NCG. b) Polarization and power density curves, c) specific capacities, and d) charge–discharge curves of ZABs. e,f) Density of state (DOS) of Fe_3_O_4_@NCG and Fe_3_O_4_/Eu_2_O_3_@NCG catalysts. a–f) Reproduced with permission.^[^ ^79^ ^]^ Copyright 2022, American Chemical Society. g) Schematic illustration of the synthesis process of NiCo_2_O_4_@NiMn LDH on Ni foam. h–k) HRTEM images of NiCo_2_O_4_ nanowires (h,i) and NiCo_2_O_4_@NiMn LDH. (j,k). g–k) Reproduced with permission.^[^ ^80^ ^]^ Copyright 2018, The Royal Society of Chemistry.

On the other hand, the rare‐earth metal oxide modification strategy has been applied to construct highly efficient and ultrastable electrodes for FSZABs.^[^ ^79^ ^]^ Hence, an Fe_3_O_4_/Eu_2_O_3_@NCG (denoting GO‐doped carbon layer as NCG) electrode was synthesized by employing layered Fe–Eu MOF/GO (Fe–Eu‐MOF/GO) as a precursor (**Figure** **12**a). A solid‐state ZAB based on such a catalyst delivered a high peak power density and energy density of 92.7 mW cm^−2^ (Figure 12b) and 854 W h kg^−1^ (Figure 12c), respectively, and excellent charge–discharge cycling stability for 460 h (Figure 12d). Based on their DFT calculation to comprehend the origin of the outstanding activity of this catalyst, the d‐band center of Fe_3_O_4_/Eu_2_O_3_@NCG (−5.365 eV) was located further away from the Fermi level than that of Fe_3_O_4_@NCG (−4.548 eV) (Figure 12e,f), which could promote the desorption of O_2_. In addition, the density of the state value was larger, suggesting a fast electron transfer rate.^[^ ^79^ ^]^

Moreover, layered double hydroxides (LDHs) usually display superior OER activity due to their large interlayer spacing for mass transport to promote the oxygen reaction. Consequently, combining LDHs with transition metal oxides to construct self‐supported bifunctional composite air electrodes is an attractive approach. Zhang and co‐workers^[^ ^80^ ^]^ prepared a NiCo_2_O_4_@NiMn LDH core–shell array on Ni foam by the deposition–calcination–deposition processes (Figure 12g). The TEM and HRTEM images were applied to characterize the morphology difference between NiCo_2_O_4_ and NiCo_2_O_4_@NiMn LDH. Unlike the pure NiCo_2_O_4_ nanowires (Figure 12g,h), NiMn LDH nanoflakes uniformly covered the NiCo_2_O_4_ nanowires surface in NiCo_2_O_4_@NiMn LDH core–shell array (Figure 12i,j). With this unique structure, high active surface area, rapid mass/charge transport, and excellent electronic conductivity, the FSZAB employing this catalyst exhibited outstanding mechanical properties, long cycle durability, and high round‐trip efficiency (70–74%).

## 4 Synthesis Strategies

Here, we summarize a few critical features that an ideal self‐supported air electrode must have for FSZABs: 1) high electronic conductivity, which is generally achieved through the conductive substrate and the large, intimate interfacial contact between the conductive substrate and the catalysts; 2) high specific surface area and abundant pores for exposure to the active sites and ensuring the efficient accessibility to oxygen and the electrolyte; 3) chemical and physical stabilities such that the electrode material is not prone to oxidation and decomposition during the testing process, ensuring the long‐term charge–discharge cycling stability for the battery; 4) the uniform dispersion of catalyst on conductive substrate surface with a tight contact; 5) novel nanostructure and morphology to achieve the hydrophobicity for the air electrode; 6) superior ORR and OER activity and stability; and finally 7) the robust contact between catalyst and substrate that enables fast electron transfer, excellent long‐term durability and flexibility under battery charging–discharging cycles.

As a result, different preparation methods have been developed and applied to develop such highly active and stable self‐supported air electrodes for FSZABs. Moreover, different catalytically active substances have also been extensively studied and prepared. Here, we listed a few common synthesis strategies for preparing self‐supported air electrodes, which include pyrolysis, hydrothermal/solvothermal, electrospinning techniques, vapor deposition, and combination of these methods.

### 4.1 Pyrolysis

**Figure 13:**
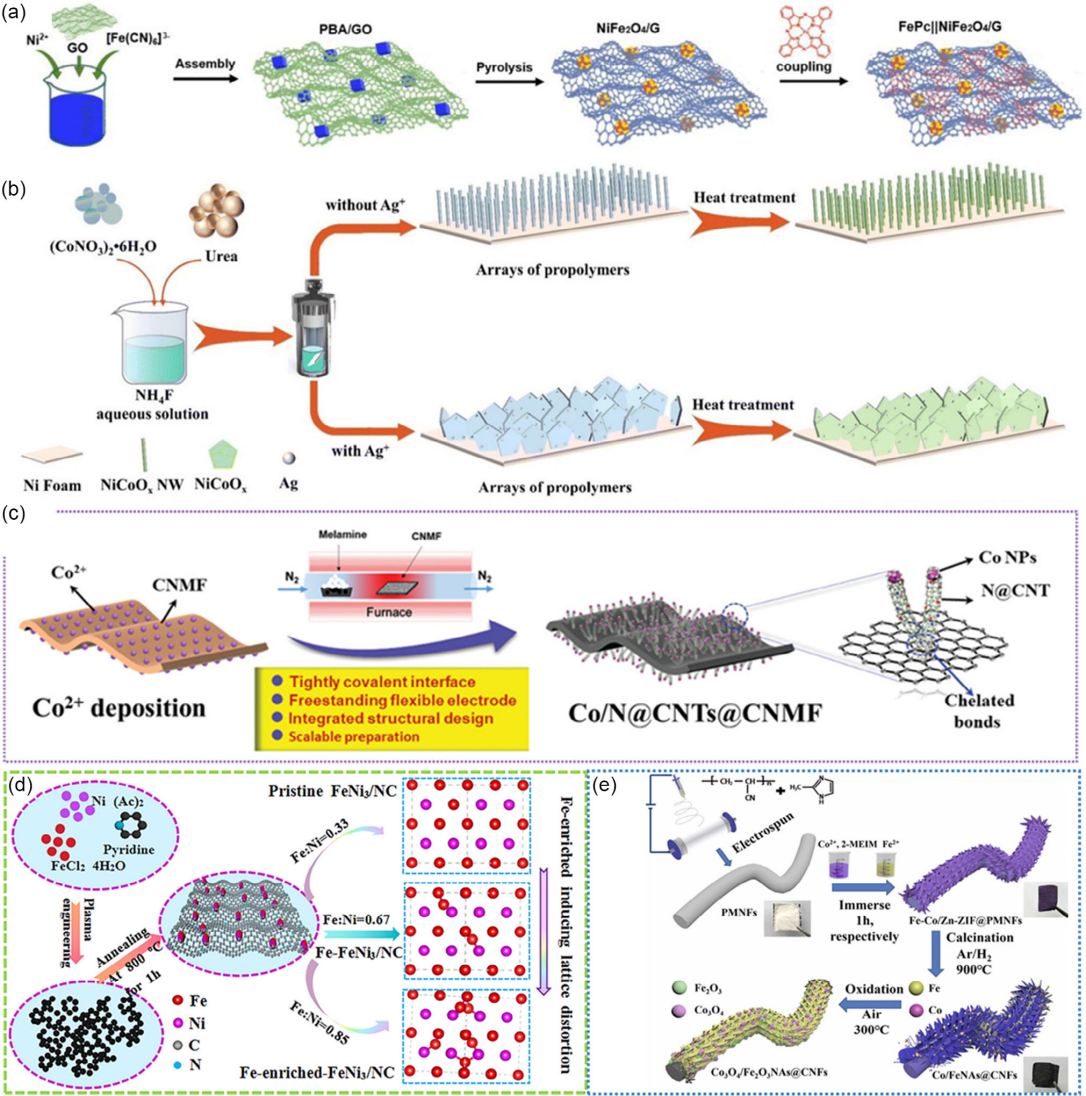
a–e) Schematic illustrations of the synthesis process of the self‐supported air electrodes for FSZABs via: a) pyrolysis, b) hydrothermal, c) chemical vapor deposition, d) physical vapor deposition, and e) electrospinning. a) Reproduced with permission.^[^ ^81^ ^]^ Copyright 2023, Elsevier B.V. b) Reproduced with permission.^[^ ^83^ ^]^ Copyright 2023, Royal Society of Chemistry. c) Reproduced with permission.^[^ ^49^ ^]^ Copyright 2020, Wiley‐VCH. d) Reproduced with permission.^[^ ^89^ ^]^ Copyright 2020, Elsevier. e) Reproduced with permission.^[^ ^74^ ^]^ Copyright 2022, Elsevier.

The pyrolysis process generally requires pregrowth of the catalysts on the conductive substrate via the in situ growth, self‐assembly, and freeze‐drying processes, which ensure the construction of the catalyst/substrate interface and prevent the agglomeration of the catalysts. Subsequently, such substrates loading catalysts are heated at a specific temperature (600–900 °C) in the presence of carbon and heteroatomic sources (N, B, P, and S), thus forming self‐supported air electrodes.^[^ ^36^, ^39^, ^41^, ^77^ ^]^ For example, a self‐supported air electrode iron phthalocyanine||NiFe_2_O_4_/G (FePc||NiFe_2_O_4_/G) was obtained by pyrolysis, and a self‐assembly strategy was used to pregrow the Prussian blue analogue (PBA) catalyst on the substrate GO surface (as carbon sources and metal sources).^[^ ^81^ ^]^ This precatalyst was further converted to FePc||NiFe_2_O_4_/G catalyst during the subsequent pyrolysis and coupling process (**Figure** **13**a).

In addition, single‐atom (SA) metal or alloy catalysts/substrates developed by metal and heteroatom doping usually display excellent ORR and OER performances. Such air electrodes can also be prepared via pyrolysis, where MOFs, ZIFs, and COFs materials are often used as pyrolysis precursors.^[^ ^36^, ^79^ ^]^ The performances of pyrolysis‐made single‐atom metal or alloy catalysts/substrates, such as Co–N_*x*_/C/Ti, CoNi alloy NCNSAs/CC, and SAFe‐SWCNT^[^ ^57^ ^]^ were explored recently. The tight contact between the catalysts and conducting substrates can facilitate electron transfer and improve the dispersion of the catalyst, thus facilitating the reaction process.

### 4.2 Hydrothermal/Solvothermal

Despite their identical working principle, if the reaction medium used is water, the process is called hydrothermal. On the other hand, if organic solvent is used as the reaction medium, the process is termed solvothermal. The reaction temperature may vary between 80 and 250 °C, giving a high temperature and pressure environment within a sealed Teflon‐lined autoclave, which allows the metal ions in the solution to recrystallize on the conductive substrate surface to obtain the self‐supported air electrodes. Generally, air electrodes with different nanostructures and morphologies can be obtained by changing the metal ions, polar functional groups, and conductive substrates in the hydrothermal/solvothermal process. The uniform growth of the catalyst on the conductive substrate surface can increase the contact tightness, thus ensuring good electrical conductivity and mechanical stability.^[^ ^82^ ^]^

Hydrothermal/solvothermal processes have the advantages of mild reaction, low emission, and high catalyst purity. Moreover, hydrothermal/solvothermal process can also be combined with pyrolysis to make the ideal air electrodes (Figure 13b).^[^ ^83^ ^]^ Researchers have recently developed self‐supported air electrodes, such as NiCoO_*x*_/NF,^[^ ^83^ ^]^ MPZCC@CNT,^[^ ^46^ ^]^ Co_3_O_4_@NiFe LDH/NF,^[^ ^45^ ^]^ NiO@Co_3_S_4_/NF,^[^ ^84^ ^]^ CoN‐Nd/CC,^[^ ^85^ ^]^ and NF/Cu_0.76_Co_2.24_O_4_/FeCo hydroxides (NF/CCO/FCH),^[^ ^86^ ^]^ all of which exhibited excellent ORR, OER, and battery performances. For example, Yang et al.^[^ ^86^ ^]^ obtained an excellent air electrode via a two‐step hydrothermal process. The FCH nanosheets formed in the second hydrothermal step uniformly modified the thin CCO nanosheets to obtain NF/CCO/FCH. Because of its unique heterogeneous structure, large three‐phase interfacial area, multiple reaction sites, and excellent electron transfer capability, the air electrode yielded a low Δ*E* 0.66 V. The resultant FSZAB displayed a power density of 35.2 mW cm^−2^, an OCV of 1.40 V, an initial round‐trip efficiency of 71.6%, and an excellent cycling stability.

### 4.3 Vapor Deposition

The vapor deposition strategy mainly includes chemical vapor deposition (CVD) and physical vapor deposition (PVD). The CVD process involves the chemical decomposition reaction of precursor materials (including metallic materials and heteroatomic sources) under high temperatures (800–1000 °C) to form gaseous products, which are subsequently deposited onto the surface of conductive substrates to give self‐supported air electrodes (Figure 13c).^[^ ^49^ ^]^ So far, the researchers have prepared various metal/CNTs as self‐supported air electrodes via CVD by utilizing the catalytic effect of transition metal nanoparticles (Co, Fe, and Ni) for the growth of CNTs under high temperatures (700–900 °C),^[^ ^87^ ^]^ such as Co/N@CNTs@CNMF,^[^ ^49^ ^]^ FeNi@NCNT@CC,^[^ ^52^ ^]^ CoO@PCNAs@CC (denoting porous carbon nanosheets arrays as PCNAs),^[^ ^88^ ^]^ and NP‐VANCT‐GF (denoting vertically aligned CNTs as VANCTs).^[^ ^65^ ^]^ The FSZABs using them as self‐supported air electrodes all yielded high power and energy densities and exhibited excellent charge–discharge cycling stability.

Unlike the CVD, the PVD process generally does not involve chemical reactions. Several methods, such as plasma engineering and magnetron sputtering, can be used to obtain the electrodes. Plasma engineering is a special plasma–liquid system that applies excited electrons and free radicals for rapid material synthesis. The utilized synthesis speed could reach about 10 mg min^−1^, thus having an enormous application potential.^[^ ^54^, ^89^ ^]^ For example, Chen et al.^[^ ^89^ ^]^ designed and prepared Fe‐enriched FeNi_3_ intermetallic nanoparticle/N‐doped carbon (Fe‐enriched FeNi_3_/NC) electrode based on plasma engineering (Figure 13d). The excess Fe ions induce a high degree of lattice distortion in this electrode, which results in abundant oxygen defects and promotes a higher active electron density around the Fermi energy level. As a result, the catalyst Fe‐enriched FeNi_3_/NC outperformed the benchmark 20 wt% Pt/C + Ir/C electrocatalyst with low potential difference (Δ*E* = 0.80 V), low charge–discharge voltage gap (0.89 V), high peak power (89 mW cm^−2^), as well as superior specific capacity of 734 mAh g_Zn_^−1^ at a 20 mA cm^−2^.

### 4.4 Electrospinning

This technique is generally combined with carbonization process to obtain self‐supported air electrodes for FSZABs. (Figure 13e).^[^ ^74^ ^]^ First, the electrostatic repulsion between surface charges manifested into the production of 1D nanofibers from polymer solutions. The morphologies and sizes of these nanofibers can be constructed by changing the composition and content of transition metal ions and dopants in polymer solutions. Then, the subsequent processes, such as carbonization and/or oxidation and/or heteroatom (N, B, P, and S) doping, can be applied to obtain self‐supported air electrodes with different structures (core–shell, hollow, and solid structures) and compositions with heteroatom doping (single‐atom, oxide, and sulfide).^[^ ^68^, ^69^ ^]^

This strategy can be applied to modulate the composition, morphology, conductivity, specific surface area, and electronic structure of the electrodes, ensuring large specific surface area, abundant porosity, multiple active sites, and excellent mechanical strength, thus improving the energy density, cycling stability, and flexibility of ZABs.^[^ ^59^, ^76^ ^]^ The single‐atom catalysts/conductive substrates prepared via electrospinning tend to possess high electrochemical activity and durability.^[^ ^56^, ^60^ ^]^ For example, the air electrode Co SA@NCF/CNF synthesized by the electrospinning–impregnation–carburization processes could yield a half‐wave potential of 0.88 V for ORR and an overpotential of 0.4 V for OER. Furthermore, the FSZAB based on Co SA@NCF/CNF could yield a specific capacity of 796 mAh g_Zn_^−1^, an energy efficiency of 67.57%, and good stability. Such superior performance could be ascribed to the atomic‐level dispersion of Co, the hierarchical porous structure, and the construction of a CNT, which guarantees the active site's accessibility and improves the electrodes’ mechanical stability.^[^ ^59^ ^]^

## 5 Summary and Outlook

By virtue of high energy density, mechanical flexibility, low cost, and high safety, FSZABs will play a key role in flexible electronics application. Developing and designing robust self‐supported bifunctional air electrodes is essential to the achieve high energy efficiency and good cycling stability in FSZABs. Electrocatalytic reactions in air electrodes involve the opposite ORR and OER processes, where O_2_ diffusion, electron transfer, ion transport, and catalyst deactivation occur concurrently. An ideal self‐supported air electrode should have enhanced electrical conductivity, abundant active sites, superior stability, and mechanical flexibility. Significant progresses in the design of such electrodes have been made recently that translate to the improvement of performance and durability of FSZABs. At the same time, arrays of advanced characterization techniques have also been employed to investigate the origin of the electrochemical activity. Furthermore, the appropriate synthesis strategy is also vital in developing self‐supported air electrodes. Various processes, i.e., pyrolysis, hydrothermal, chemical vapor deposition, physical vapor deposition, and electrospinning, are available, which provide pathways to create unique catalysts’ structure, morphology, size, and dispersity. Such tailoring, in turn, allows adjustment of the electrochemical activity and durability of the air electrodes.

This review highlights the recent development of typical self‐supported bifunctional air electrodes (metal‐free carbon materials, transition metals/conductive substrates, transition metal compounds/conductive substrates, and other air electrodes), including the structural design, performance optimization, synthesis strategies, and their application in FSZABs. Several studies have indicated that the use of self‐supported bifunctional air electrodes in FSZABs could improve the round‐trip efficiency, energy density, OCV, and cycling durability. However, the performance and long‐term cycling life of FSZABs are still far from commercialization expectation compared to aqueous ZABs. We listed below the considerable scientific and technological challenges that need to be overcome for further practical applications. 1) The performance of FSZABs at this stage still cannot meet the requirements of commercial flexible and wearable electronic devices. The low round‐trip efficiency, power density, and short cycling stability are still unsatisfactory. The development of self‐supported bifunctional air electrodes is the most critical factor that determines the ORR, OER, and battery performances. The air electrodes are composed of active catalysts and conductive substrates. The selection of active catalysts should mainly focus on intrinsic activity, stability, conductivity, and hydrophilicity/hydrophobicity. Besides, the substrates should possess high electronic conductivity, mechanical flexibility, lightweight, and anticorrosion simultaneously. The strong interaction and abundant intimate contact between catalyst and substrate ensure good long‐term durability and flexibility, suppressing the detachment of active catalysts. Furthermore, the facile and scalable synthesis strategy is also essential in lowering the cost of FSZABs. 2) Advanced theoretical calculations and characterization methods are important for exploring reaction mechanisms and performance improvement. First, DFT calculation can provide insights into the OER and ORR mechanisms and how to further enhance the electrochemical activity and stability. However, the structure of self‐supported air electrodes may be complex due to the simultaneous inclusion of catalyst and substrate, making the construction and optimization of crystal models challenging. Second, traditional characterizations have mainly focused on catalysts’ structure, morphology, electronic states, and the catalytic mechanisms for ORR and OER. However, the interaction of catalysts and substrates, their structural changes during the reaction, and their influence on the ZAB performance and durability have not been studied in detail. 3) A more systematic evaluation criterion for FSZABs is necessary for comparison. First, different indicators criteria or forms in terms of specific capacity, energy density, and/or cycle stability have been used to evaluate battery performance in the literature, making it difficult to compare them. In addition, most literature only provides battery performance at low current density (<20 mA cm^−2^), which fails to meet the requirements of practical applications (>50 mA cm^−2^). Finally, mechanical stability cannot be overlooked for the application of solid‐state ZABs in flexible devices. However, most works only show the cycling stability at some bending angles to prove the flexibility of the battery, which may not be enough to evaluate the mechanical stability and flexibility of the ZABs. 4) In addition to using self‐supported air electrodes and solid electrolytes, optimizing other components for solid‐state ZABs can also be critical for their application in wearable/foldable electronic devices. For example, the parasitic reaction between Zn and electrolytes and the formation of Zn dendrites can reduce the availability of active sites and the battery lifetime. Studies have shown that Zn anodes’ surface or composition modification can effectively resolve this dilemma. In addition, the structure and assembly technology of solid‐state ZABs is also essential for their application in practical devices.

## Conflict of Interest

The authors declare no conflict of interest.
